# Alternative splicing in human cancer cells is modulated by the amiloride derivative 3,5‐diamino‐6‐chloro‐N‐(N‐(2,6‐dichlorobenzoyl)carbamimidoyl)pyrazine‐2‐carboxide

**DOI:** 10.1002/1878-0261.12524

**Published:** 2019-06-01

**Authors:** Chien‐Chin Lee, Wen‐Hsin Chang, Ya‐Sian Chang, Jinn‐Moon Yang, Chih‐Shiang Chang, Kai‐Cheng Hsu, Yun‐Ti Chen, Ting‐Yuan Liu, Yu‐Chia Chen, Shyr‐Yi Lin, Yang‐Chang Wu, Jan‐Gowth Chang

**Affiliations:** ^1^ Epigenome Research Center China Medical University Hospital Taichung Taiwan; ^2^ Department of Primary Care Medicine Taipei Medical University Hospital Taiwan; ^3^ Department of Laboratory Medicine China Medical University Hospital Taichung Taiwan; ^4^ Center for Precision Medicine China Medical University Hospital Taichung Taiwan; ^5^ TIGP‐Bioinformatics Institute of Information Science Academia Sinica Taipei Taiwan; ^6^ Institute of Bioinformatics and Systems Biology National Chiao Tung University Hsinchu Taiwan; ^7^ Department of Biological Science and Technology National Chiao Tung University Hsinchu Taiwan; ^8^ Graduate Institute of Pharmaceutical Chemistry China Medical University Taichung Taiwan; ^9^ Graduate Institute of Cancer Molecular Biology and Drug Discovery College of Medical Science and Technology Taipei Medical University Taiwan; ^10^ Department of General Medicine School of Medicine College of Medicine Taipei Medical University Taiwan; ^11^ TMU Research Center of Cancer Translational Medicine Taipei Medical University Taiwan; ^12^ Graduate Institute of Natural Products Kaohsiung Medical University Taiwan; ^13^ Research Center for Natural Products and Drug Development Kaohsiung Medical University Taiwan; ^14^ Department of Medical Research Kaohsiung Medical University Hospital Taiwan; ^15^ Chinese Medicine Research and Development Center China Medical University Hospital Taichung Taiwan

**Keywords:** alternative splicing, amiloride, apoptosis, cancer, sorafenib

## Abstract

Alternative splicing (AS) is a process that enables the generation of multiple protein isoforms with different biological properties from a single mRNA. Cancer cells often use the maneuverability conferred by AS to produce proteins that contribute to growth and survival. In our previous studies, we identified that amiloride modulates AS in cancer cells. However, the effective concentration of amiloride required to modulate AS is too high for use in cancer treatment. In this study, we used computational algorithms to screen potential amiloride derivatives for their ability to regulate AS in cancer cells. We found that 3,5‐diamino‐6‐chloro‐N‐(N‐(2,6‐dichlorobenzoyl)carbamimidoyl)pyrazine‐2‐carboxamide (BS008) can regulate AS of apoptotic gene transcripts, including *HIPK3*,* SMAC*, and *BCL‐X*, at a lower concentration than amiloride. This splicing regulation involved various splicing factors, and it was accompanied by a change in the phosphorylation state of serine/arginine‐rich proteins (SR proteins). RNA sequencing was performed to reveal that AS of many other apoptotic gene transcripts, such as *AATF*,* ATM*,* AIFM1*,* NFKB1*, and *API5*, was also modulated by BS008. *In vivo* experiments further indicated that treatment of tumor‐bearing mice with BS008 resulted in a marked decrease in tumor size. BS008 also had inhibitory effects *in vitro*, either alone or in a synergistic combination with the cytotoxic chemotherapeutic agents sorafenib and nilotinib. BS008 enabled sorafenib dose reduction without compromising antitumor activity. These findings suggest that BS008 may possess therapeutic potential for cancer treatment.

**Abbreviations**
AS: alternative splicing
DSBs: DNA double‐strand breaks
HCC: hepatocellular carcinoma
HIPK3: homeodomain‐interacting protein kinase 3
hnRNPs: heterogeneous RNPs
OA: okadaic acid
PP1: protein phosphatase‐1
PTMs: post‐translational modifications
RBPs: RNA‐binding proteins
SiMMaps: site‐moiety maps
SMAC/DIABLO: second mitochondria‐derived activator of caspases
SR proteins: serine/arginine‐rich proteins
SREs: splicing regulatory sequence elements
SRPKs: SR protein‐specific kinases

## 1 Introduction

In eukaryotes, alternative splicing (AS) is a crucial mechanism for generating transcriptome diversity and can result in single‐gene coding for multiple proteins. Currently, 20 000 coding genes are estimated to be able to produce nearly 80 000 proteins and probably many more through AS in humans (Hahn *et al*., 2015). Protein isoforms that are generated from the same gene through AS may have the same, similar, or even opposite biological properties (e.g., protein–protein interactions, subcellular localization, or catalytic ability) (Tazi *et al*., 2009). Furthermore, splicing can be modulated through the interplay between RNA‐binding proteins (RBPs) and splicing regulatory sequence elements (SREs) in mRNA. Numerous RBPs, which function to regulate AS, have been studied, and the well‐known regulators of splice site selection are serine/arginine‐rich (SR) proteins and heterogeneous RNPs (hnRNPs). In general, serine/arginine‐rich proteins (SR proteins) bind to exonic splicing enhancers through their RNA‐binding domain and promote exon inclusion by recruiting spliceosome components. By contrast, hnRNPs often bind to exonic splicing silencers or intronic splicing silencers to cause exon exclusion. SR proteins and hnRNPs generally display an antagonistic function toward each other in SRE selection (Fu and Ares, 2014). Therefore, AS can be determined by a balance between positive and negative regulation, and the modulation of RBPs may alter the final outcome of the splicing reaction. In cancer progression, the AS process is generally interrupted, leading to the creation of both functional and nonfunctional end products. Cancer cells often take advantage of these specific splicing events to produce oncogenes or aberrant tumor suppressors that promote growth and survival (Sveen *et al*., 2016; Venables, 2004). This implies that shifting splicing toward the appropriate isoform may offer therapeutic potential in cancer treatment.

Amiloride is a potassium‐sparing diuretic that was first approved for use in 1967. Its function is to help the kidneys to remove excess sodium and water and retain potassium in the body, and it is usually used to treat hypertension, heart failure, liver cirrhosis, and other diseases that result in edema and ascites (Bull and Laragh, 1968; Loffing and Kaissling, 2003). In our previous studies, we have determined a novel biological action of amiloride, namely the modulation of AS in human cancer cells. Amiloride was able to modulate the AS of various apoptotic genes, including *BCL‐X* and *homeodomain‐interacting protein kinase 3* (*HIPK3*), with a concomitant effect in many splicing factors, such as SRSF1, hnRNP A1, hnRNP A2/B1, and hnRNP C1/C2, finally resulting in apoptosis (Chang *et al*., 2011b). However, the effective concentration of amiloride is too high to limit its applications in cancer treatment. Therefore, in this study, we used computational algorithms to predict amiloride derivatives and attempted to find efficient amiloride derivatives to determine possible therapeutic applications.

## 2 Materials and methods

### 2.1 Preparation of protein structures and screening databases

To define the binding sites, the RNA/DNA‐bound structures of snRNP70 (PDB code 4PKD, Kondo *et al*., 2015), hnRNP I (PDB code 2AD9, Oberstrass *et al*., 2005), and U2AF2 (PDB code 4TU8, Agrawal *et al*., 2014) were aligned to the structure 4PKD using the structure alignment tool CE (Guda *et al*., 2004) in order to identify similar structures. The binding sites were defined by residues situated ≤ 8 Å from the bound RNA/DNA. We selected compounds from the public database of the National Cancer Institute to generate the SiMMap. The total number of selected compounds for screening was nearly 50 000.

### 2.2 Computational screening and establishment of site‐moiety maps

**Figure 1:**
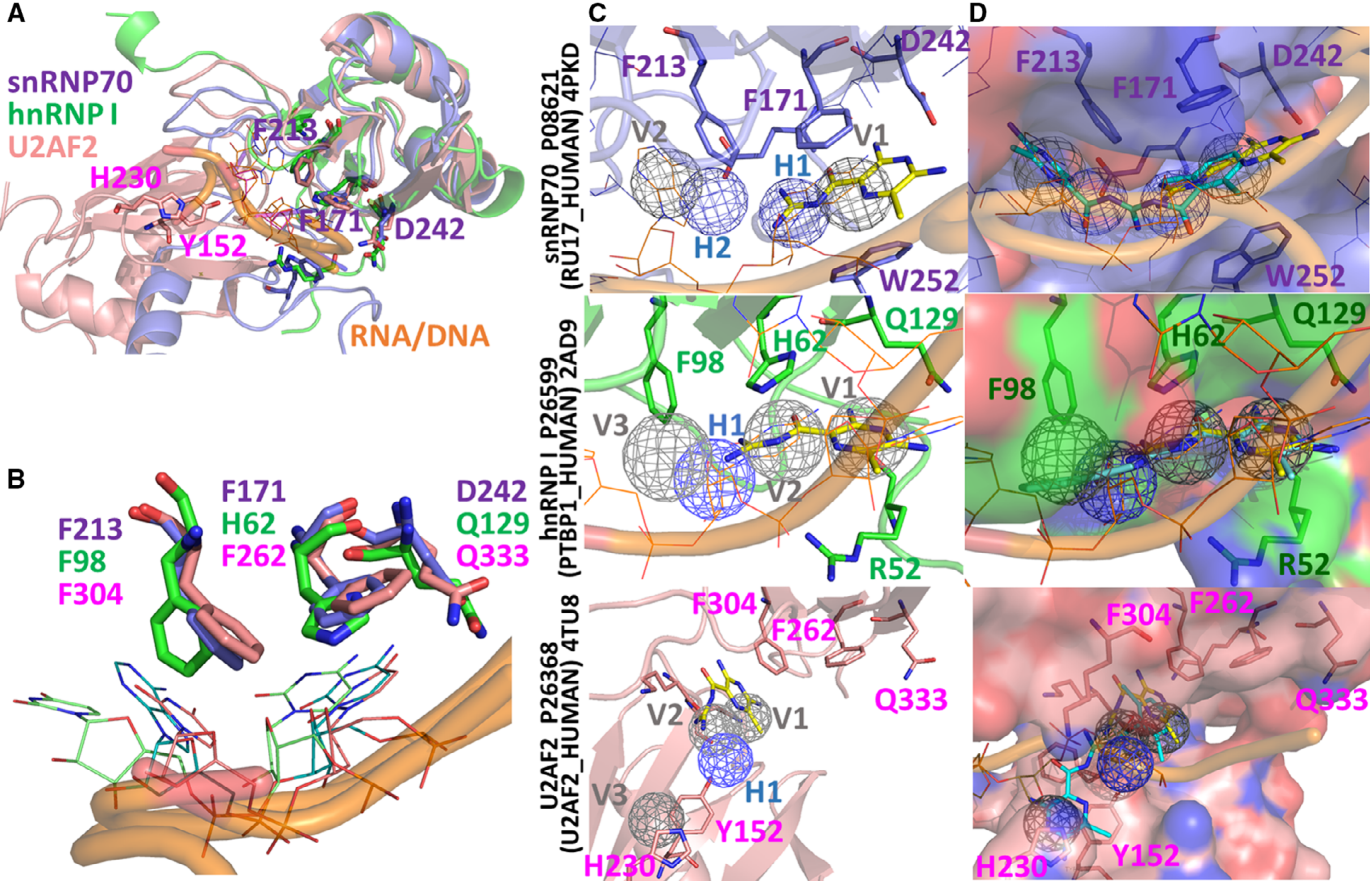
Computational results of potential target proteins for amiloride and its derivatives. (A) Structural alignments of three potential target proteins (snRNP70, hnRNP I, and U2AF2) with similar RNA/DNA binding sites. (B) The residues of snRNP70 (F213 and F171, purple), hnRNP I (green), and U2AF2 (pink) interacting with the ribose were well aligned and formed strong vdW force. (C) Docked poses (yellow) of amiloride and the SiMMaps of snRNP70, hnRNP I, and U2AF2 (pink) with anchors, H‐bond (blue), and vdW (gray). (D) Docked poses and SiMMaps of BS008 amiloride in target proteins.

**Figure 2:**
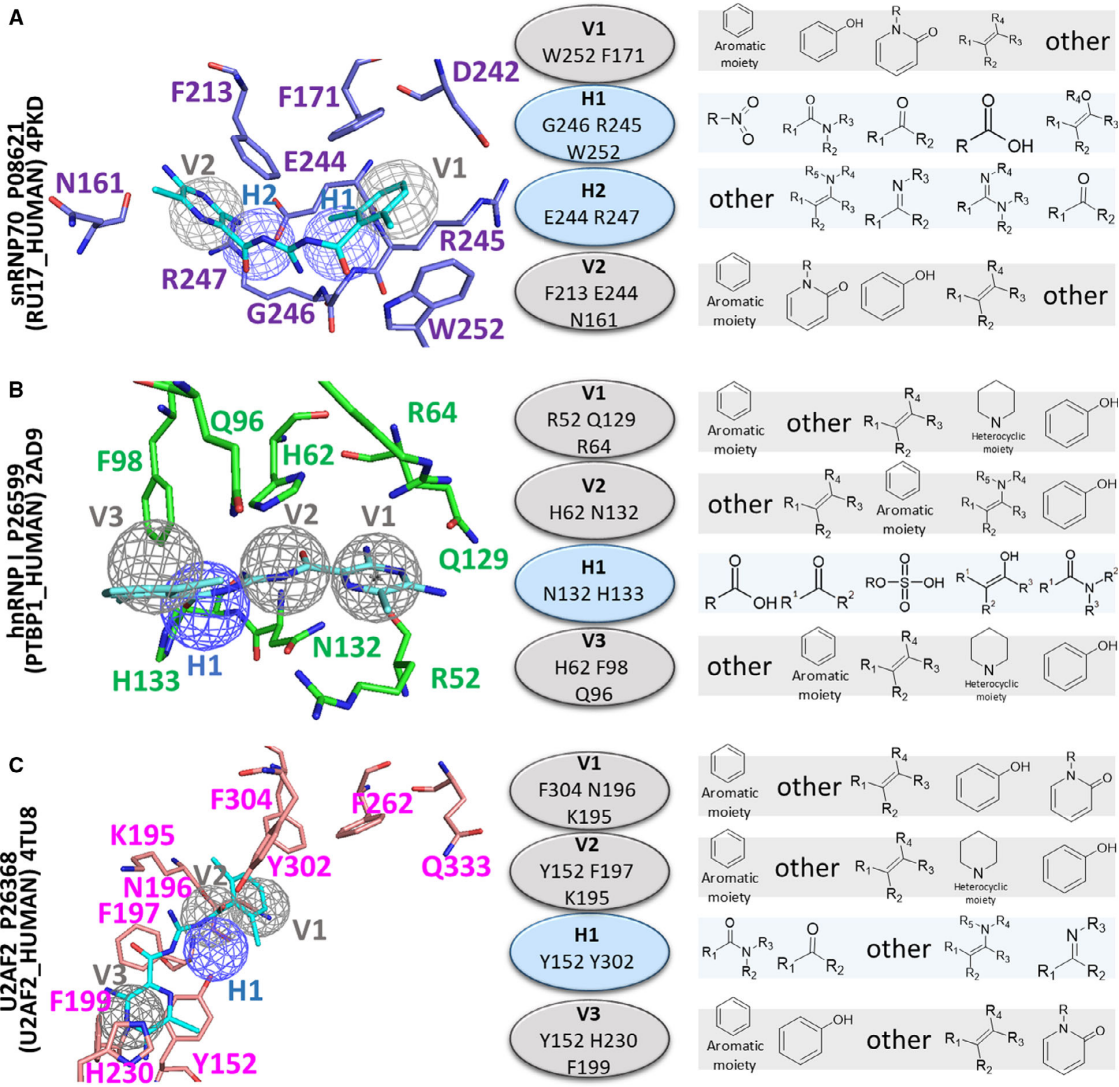
SiMMaps of potential targets. The anchors with interacting residues and moiety preferences of SiMMaps for (A) snRNP70, (B) hnRNP I, and (C) U2AF2. Hydrogen‐bonding and vdW anchors are colored in blue and gray, respectively.

The approximately 50 000 compounds were docked into the binding sites of snRNP70, hnRNP I, and U2AF2 using an in‐house docking tool, GEMDOCK (Yang and Chen, 2004), with docking parameters optimized as per virtual screening protocols. Subsequently, the top 2% of compounds (approximately 1000) with the greatest interaction energies for each protein were selected to establish the SiMMap, which described the interaction preferences between binding pockets and moieties. First, protein–compound interaction profiles were generated based on piecewise linear potential as calculated by GEMDOCK. The interaction profiles described the interactions, including electrostatic (E), hydrogen‐bonding (H), and van der Waals (vdW) interactions, between the compounds and the target protein residues. A matrix (*M*) of size *C × R* was used to present the profile, where *C* is the number of docked compounds and *R* is the number of interacting residues of a protein. An entry (*M*_*ij*_), representing the interacting energy between compound *i* and residue *j*, was considered significant if it had a *Z*‐score of ≥ 1.65. Then, spatially neighboring interacting residues and their interactive moieties with statistical significance were assigned as an anchor (Figs 1C and 2). Finally, the SiMMap of each target protein was constructed.

### 2.3 Reagents

Reagents were purchased from the following companies: amiloride, Dulbecco's modified Eagle's medium (DMEM), FBS, penicillin, and streptomycin from Gibco‐BRL (Grand Island, NY, USA); AKT inhibitor from Millipore (Billerica, MA, USA); and 3‐(4,5‐dimethylthiazol‐2‐yl)‐2,5‐diphenyltetrazolium bromide (MTT), DMSO, Kolliphor EL, nilotinib, OA, propidium iodide (PI), and sorafenib from Sigma‐Aldrich (St. Louis, MO, USA).

### 2.4 Antibodies

Antibodies were purchased from the following companies: anti‐H3K27me1, anti‐H3K27me3, anti‐H3K79me1, anti‐H3K79me2, anti‐histone H3, anti‐hnRNP I, anti‐KDM4A, anti‐KDM5A, anti‐KDM5C, anti‐snRNP70, anti‐tubulin, and anti‐U2AF2 from Abcam (Cambridge, MA, USA); anti‐hnRNP A2/B1 from Acris (San Diego, CA, USA); anti‐SR protein‐specific kinases (SRPK)1 and anti‐SRPK2 from BD Biosciences (San Diego, CA, USA); anti‐AKT, anti‐H3K27me2, anti‐protein phosphatase‐1 (PP1), anti‐phospho‐PP1 at Thr320, anti‐phospho‐AKT at Ser473, and anti‐cleaved‐CASPASE‐3 from Cell Signaling Technology (Billerica, MA, USA); anti‐H3K4me1, anti‐H3K4me2, anti‐H3K4me3, and anti‐H3K36me3 from Millipore; anti‐pro‐CASPASE‐3 and anti‐KDM7A from GeneTex (SanAntonio, TX, USA); anti‐hnRNP C1/C2, anti‐BAX, and anti‐BCL2L1 from Santa Cruz Biotechnology (Santa Cruz, CA, USA); anti‐hnRNP A1 from Sigma; and anti‐SRSF1 and anti‐SRSF3 from Zymed (San Francisco, CA, USA).

### 2.5 Cell culture and gene knockdown

A549, LoVo, and Huh‐7 cell lines were obtained from the Bioresource Collection and Research Center, Taiwan. Ba/F3 transfectant (expressing Bcr‐Abl with kinase domain point mutations T315I) was provided by Michael W. Deininger (O'Hare *et al*., 2005). Cells were maintained in DMEM (solid tumor‐cell lines) and RPMI‐1640 medium (leukemia cell lines) supplemented with 10% FBS and antibiotics (100 U·mL^−1^ penicillin and 100 μg·mL^−1^ streptomycin) at 37 °C in a humidified atmosphere of 5% CO_2_. To perform snRNP70 or hnRNP I knockdown, antisense oligonucleotide sequences of snRNP70, hnRNP I, or a scrambled control (Table S1) were transfected into cells using Lipofectamine RNAiMAX (Invitrogen, Carlsbad, CA, USA) according to the manufacturer's protocol.

### 2.6 Cell growth inhibition assay

Cells were prepared in a 96‐well plate, and 10 μL of MTT solution (5 mg·mL^−1^) was added to each well. After 2 h, the medium was removed and cells were lysed with 100 μL of DMSO. The absorbance at 565 nm was measured using a microplate reader, and the results are presented as a percentage of the control results.

### 2.7 Protein extracts and western blotting

Proteins were extracted using a cell lysis solution (20 mm Tris/HCl, pH 7.5, 150 mm NaCl, 1 mm EDTA, 1% Nonidet P‐40, 1% sodium deoxycholate, 2.5 mm sodium pyrophosphate, 1 mm b‐glycerophosphate, 1 mm Na_3_VO_4_, and 1 μg·mL^−1^ leupeptin) and separated by sodium dodecyl sulfate/polyacrylamide gel electrophoresis (SDS/PAGE). After proteins were transferred onto polyvinylidene difluoride membranes (Millipore), the membranes were blocked with 5% BSA (Santa Cruz Biotechnology) and then exposed at 4 °C overnight to the indicated primary antibodies, followed by horseradish peroxidase‐conjugated secondary antibody for detection by an ECL chemiluminescence detection system (GE Healthcare, Pittsburgh, PA, USA).

### 2.8 Reverse transcription PCR analysis

Next, mRNA was obtained using a TurboCapture 8 mRNA kit (Qiagen, Chatsworth, CA, USA) and then converted into cDNA using High‐Capacity cDNA Reverse Transcription Kits (Applied Biosystems) according to the manufacturer's instructions. PCR was performed using specific pairs of primers (Table S2).

### 2.9 Cell cycle distribution

Cells were collected and fixed in 70% (v/v) ethanol at 4 °C overnight and then stained with 1 mL of PI staining buffer (0.1% Triton X‐100, 100 μg·mL^−1^ RNase A, and 500 μg·mL^−1^ PI in phosphate‐buffered saline) at 37 °C for 30 min. Data were collected and analyzed using a BD FACSAria™ Fusion flow cytometer (BD Biosciences).

### 2.10 RNA sequencing

Samples were prepared using an mRNA‐seq sample kit (Illumina, San Diego, CA, USA) according to the manufacturer's instructions. The raw sequencing data were deposited in the National Center for Biotechnology Information's Gene Expression Omnibus (GEO), and they can be obtained through GEO Series with the accession number GSE110059.

### 2.11 Huh‐7 xenograft model

BALB/cAnN.Cg‐Foxn1^nu^/CrlNarl mice (male, 4–6 weeks old) were purchased from the National Laboratory Animal and Research Center (Taipei, Taiwan); 5 × 10^6^ Huh‐7 cells were subcutaneously injected into the right flank of the mice. Therapy started after 3–4 weeks when the tumors had reached an average volume of 300 mm^3^. The mice were assigned to groups of six and subcutaneously injected daily with 0.1 mL of solvent (final volume ratio of DMSO/Kolliphor EL/PBS was 4.5 : 4.5 : 91) for the control group or 30.9 μg/10 g of BS008 for the treatment group. The longest axis (a), shortest axis (b), and thickness (c) of the tumor were measured with calipers, and the tumor volume (V) was evaluated using the formula V = πabc/6. All animal experiments were performed in accordance with the guidelines established by the Institutional Animal Care and Use Committee (IACUC) of China Medical University (CMU). All animals were housed in the Laboratory Animal Center of CMU under a 12‐h light/dark (08:00/20:00) cycle with free access to food and water. The mice were sacrificed using CO_2_, and the tumor tissues were subsequently harvested. All breeding and subsequent use of animals in this study, including sacrifice, was approved by the IACUC of CMU (approval number 103‐264‐B).

### 2.12 Statistical analysis

Differences between the control and treatment were analyzed using Student's *t*‐test, with a probability of less than 5% (*P *<* *0.05) considered significant.

## 3 Results

### 3.1 Identification of target proteins

We applied Homopharma (Chiu *et al*., 2014), similar compound structures often targeting proteins with similar binding sites and sharing interactions, to identify potential target proteins of amiloride. First, we collected known target proteins (Table S3) of amiloride and proteins (Fu and Ares, 2014) that are relative to the functions/pathways of AS (Table S4). Subsequently, we searched the similar compound structures of amiloride and their target proteins recorded in the BindingDB database (Gilson *et al*., 2016). On the basis of these proteins, we used a structure alignment tool (CE, Guda *et al*., 2004) to identify the proteins with similar binding environments, such as snRNP70 (PDB code 4PKD), hnRNP I (PDB code 2AD9), and U2AF2 (PDB code 4TU8) (Fig. 1A). Their root‐mean‐square derivations were 1.14 and 1.35 Å when hnRNP I and U2AF2 aligned with snRNP70, respectively. Notably, these three proteins were cocrystalized with mRNA, and their aromatic amino acids (F213 and F171 in snRNP70; F98 and H62 in hnRNP I; and F304 and F262 in U2AF2) consistently formed strong vdW contacts with the rings of RNA (Agrawal *et al*., 2014; Kondo *et al*., 2015; Oberstrass *et al*., 2005) (Fig. 1B).

After identifying these potential target proteins, we integrated molecular docking (GEMDOCK, Yang and Chen, 2004) and statistically analyzed thousands of docked poses for site‐moiety maps (SiMMaps, Chen *et al*., 2010) to further investigate and evaluate their binding environments. Our previous studies have shown that GEMDOCK has a similar performance to other docking methods (Ewing *et al*., 2001; Kramer *et al*., 1999) and was successfully used to identify novel inhibitors and binding sites (Chin *et al*., 2010; Hsu *et al*., 2013, 2015; Yang *et al*., 2007). An anchor of a SiMMap possesses three properties: a binding subpocket with interacting residues, the moiety composition of screening compounds, and the pocket–moiety interaction type (electrostatic, hydrogen‐bonding, or vdW). In this study, we docked approximately 50 000 compounds collected from the public database of the National Cancer Institute using GEMDOCK. Subsequently, the SiMMaps of snRNP70, hnRNP I, and U2AF2 were established by statistically analyzing the top 1000 docked compounds (approximately 2% of the 50 000 compounds; Figs 1C and 2). The SiMMaps of snRNP70 (four anchors) and hnRNP I (four anchors) are similar, and their anchors can be well aligned. Therefore, the anchors V1, H1, H2, and V2 of snRNP70 were aligned to the anchors V1, V2, H1, and V3 of hnRNP I, respectively. The docked aromatic moiety of amiloride occupied the anchor (V1 in snRNP70 and V1 in hnRNP I) and imitated the ribose of RNA, forming π–π vdW with the rings of F171 and W252 in snRNP70 as well as residues H62 and R52 in hnRNP I. Conversely, the SiMMap position of U2AF2 differs from that of snRNP70 and hnRNP I because the aromatic residues of these three proteins consistently form strong vdW forces with the ribose of RNA (Fig. 1B,C). These results indicate that a SiMMap provides a comprehensive analysis and its anchors describe the relationship between the moiety preferences and physicochemical properties of the binding site, as determined by the interaction profiles between target proteins and docked compounds.

### 3.2 Site‐moiety map for amiloride optimization

Based on the SiMMaps of these three target proteins, we utilized their anchor distributions and moiety preferences to guide the lead optimization of amiloride (Figs 1C and 2). The aligned SiMMap of snRNP70 and hnRNP I showed that amiloride occupied two anchors in snRNP70 (V1 and H1) as well as in hnRNP I (V1 and V2) (Fig. 2). The positions and moiety preferences of the two nonoccupied anchors (i.e., V2 and H2 in snRNP70) in the aligned SiMMap could be utilized for designing amiloride derivatives and improving its potency. For example, the majority of the 550 compounds (approximately 55%) located in the V2 anchor formed vdW forces with anchor residues (F213, E244, and N161 in snRNP70) through their ring‐based moieties. To design amiloride derivatives, a dichlorobenzene was considered as a potential moiety to mimic the ribose of RNA to form vdW and hydrogen bonds with anchor residues (F213, E244, and N161 in V2 in snRNP70; H62, F98, and Q96 in V3 in hnRNP I). The docked poses (blue) of 3,5‐diamino‐6‐chloro‐N‐(N‐(2,6‐dichlorobenzoyl)carbamimidoyl)pyrazine‐2‐carboxamide (BS008) occupied four anchors in snRNP70 and hnRNP I (Fig. 1D). For the H2 anchor in snRNP70 and V2 in hnRNP I, the anchor residues (E244 and R247 in snRNP70; H62 and N132 in hnRNP I) formed vdW and hydrogen‐bonding forces with the carboxamide moiety of BS008. According to the aligned SiMMap, U2AF2 lost aligned anchors (i.e., H1 and V1 in snRNP70) to form a sandwich conformation interacting with BS008. These results show that BS008 inhibited both snRNP70 and hnRNP I but not U2AF2 in validation *in vitro*.

### 3.3 Effect of BS008 on cell viability

**Figure 3:**
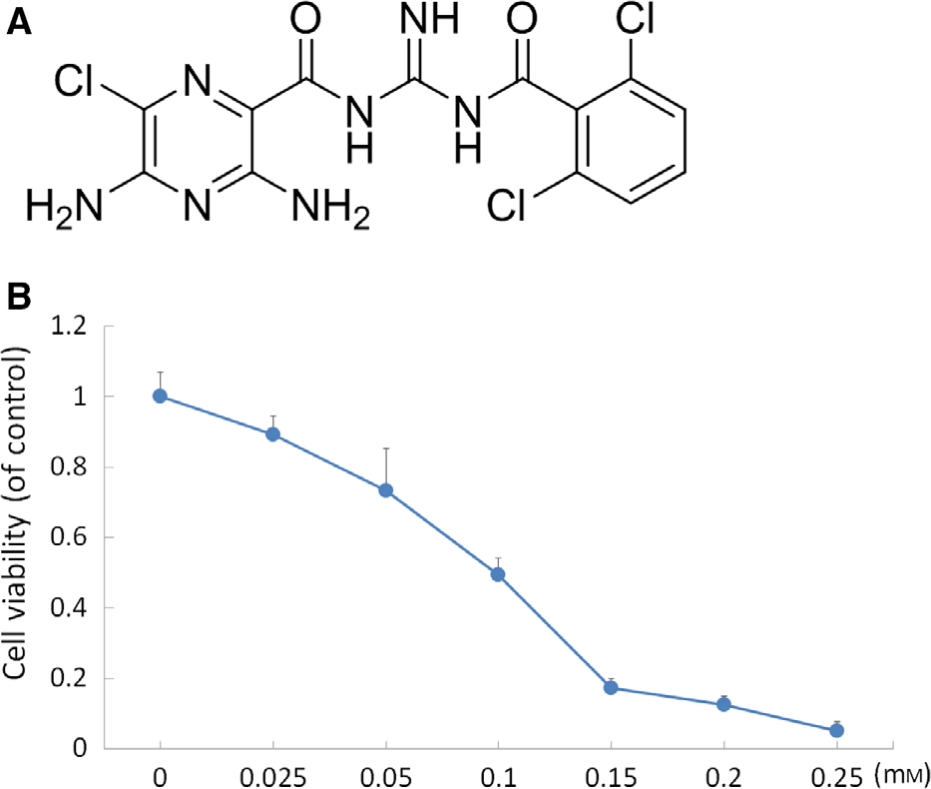
Effect of BS008 on the viability. (A) Chemical structures of BS008. (B) Huh‐7 cells were treated with BS008 at the indicated concentrations for 24 h, and the viability was analyzed using MTT assay. Data are presented as mean ± standard deviation (SD) from three independent experiments.

According to the predictions of the computational algorithms, BS008 (Fig. 3A) was synthesized and provided by Yang‐Chang Wu (Graduate Institute of Natural Products, Kaohsiung Medical University, Kaohsiung, Taiwan). First, we used an MTT assay to assess the growth‐inhibitory activity of BS008 in human hepatocellular carcinoma (HCC) Huh‐7 cells. Huh‐7 cells were treated with various concentrations of BS008 for 24 h, and the results showed that the BS008 treatment exhibited a dose‐dependent loss of cell viability, with the half maximal inhibitory concentration (IC_50_) of BS008 being 0.128 mm (Fig. 3B). This inhibitory effect of BS008 on cell survival demonstrated in the Huh‐7 cells implied that BS008 might have potential for use in cancer treatment.

### 3.4 BS008 regulates the AS of apoptotic gene transcripts

**Figure 4:**
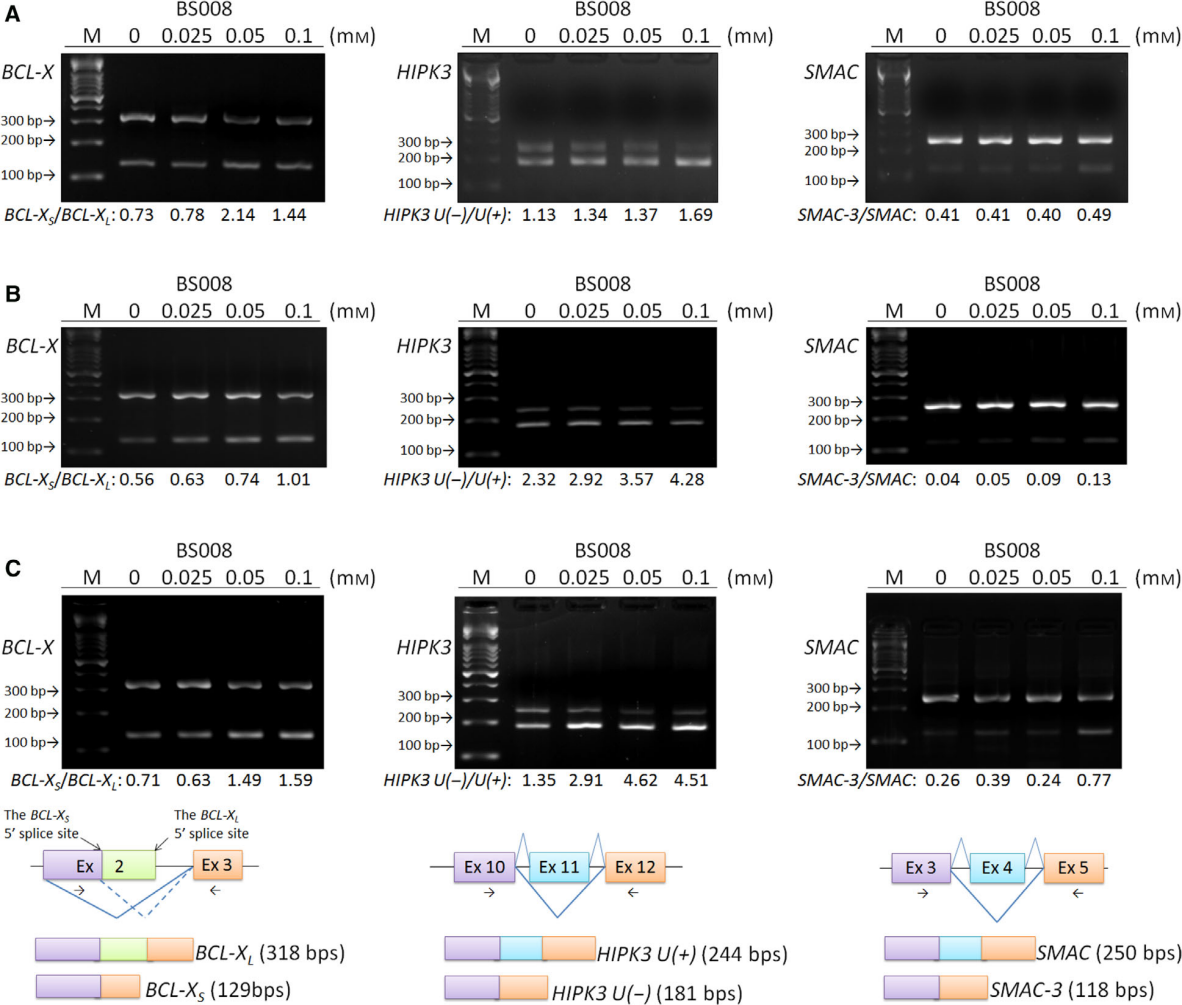
BS008 modulates the AS of apoptotic gene transcripts. Cells were treated with BS008 at the indicated concentrations for 24 h. mRNA was extracted and detected using RT‐PCR for the AS of *BCL‐X*, *HIPK3*, and *SMAC* transcripts in (A) Huh‐7, (B) A549, and (C) LoVo cells. The splicing isoforms are illustrated in the bottom row, and their expected PCR products derived through the primers are indicated by arrowheads. M, marker.

The abnormal AS of apoptotic gene transcripts has been reported to possibly be connected to carcinogenesis (Lin, 2017). Since we have previously reported that amiloride regulates the AS of *BCL‐X* and *HIPK3* pre‐mRNA in various cancer cell lines including human HCC cell line Huh‐7 (Chang *et al*., 2011a,2011b), it would be of interest to investigate if BS008 had the same effects on AS in Huh‐7. Due to the importance of *second mitochondria‐derived activator of caspases* (*SMAC/DIABLO*) in determining the sensitivity of cancer cells to apoptotic death, it was also chosen to investigate whether its splicing variants could be regulated by BS008. Huh‐7 cells treated with BS008 showed a decrease of the antiapoptotic splice variants, *BCL‐X*_*L*_ and *HIPK3 U*(*+*), while relative maintenance of the proapoptotic splice variants *BCL‐X*_*S*_ and *HIPK3 U*(*−*). Western blot analysis further confirmed a reduced level of BCL‐X_L_ protein after the treatment with BS008 (Fig. 9D). Besides *BCL‐X* and *HIPK3*, we also found *SMAC* was regulated by BS008 with an increase in the proapoptotic splice variant *SMAC‐3* (Fig. 4A). Similar splicing patterns were also observed in A549 lung cancer cells and LoVo colon cancer cells (Fig. 4B,C). We also found that BS008 exerts an effect on AS of these gene transcripts in K562 leukemia cells by detecting an increase in the proapoptotic splice variants *BCL‐X*_*S*_, *SMAC‐3,* and *HIPK3 U*(−) with a concomitant decrease in the antiapoptotic splice variant *HIPK3 U*(*+*). Notably, using highly imatinib‐resistant BaF3/Bcr‐Abl T315I cells, we observed that BS008 slightly increased the proapoptotic splice variants *BCL‐X*_*S*_ and *SMAC‐3*, but not *HIPK3 U*(−). On the contrary, we detected no alteration in the *BCL‐X*,* HIPK3*, and *SMAC* splicing patterns in normal mononuclear cells from healthy individuals (Fig. S1).

These results suggested that BS008 might regulate the apoptosis‐related pathway through modulating the AS of apoptotic transcripts in cancer cells. Transient transfection of exon 10–12 spliced *HIPK3 U*(*+*) cDNA (Fig. S2A) had no effects on the BS008‐modulated *HIPK3* splicing. However, transient transfection of *HIPK3 U*(*+*) intron containing mRNA resulted in the protection of AS of *HIPK3 U*(*+*) regulated by BS008, which might be a cause for the competition between pre‐mRNAs for the splicing machinery (Fig. S2B). Cell viability assays further confirm that inhibition of the antiapoptotic *HIPK3 U*(*+*) splicing could partially rescue the cell death induced by BS008 (Fig. S2B).

### 3.5 BS008 affects the expression levels of SR proteins and hnRNPs

**Figure 5:**
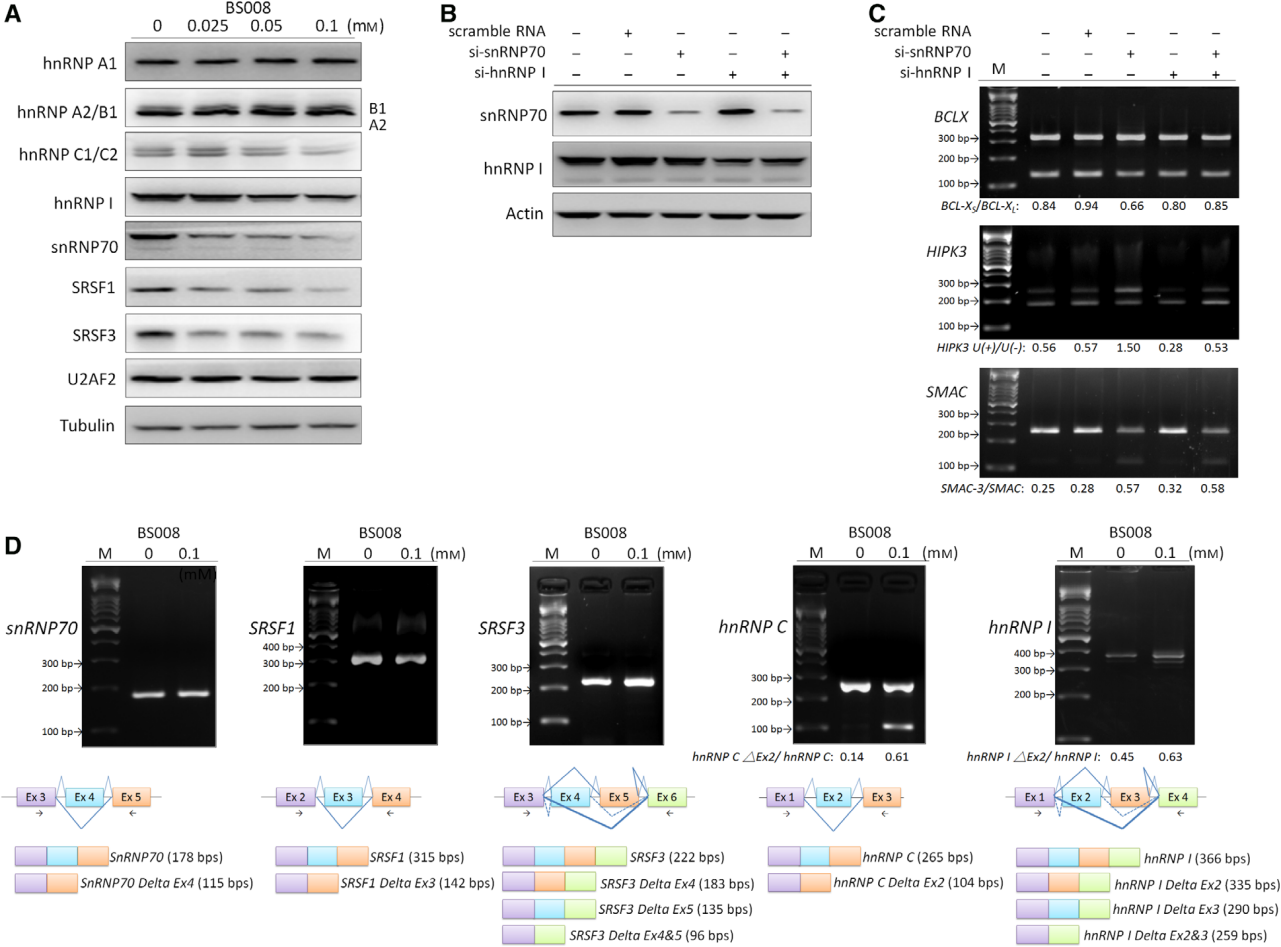
Effects of BS008 on SR proteins and hnRNPs. (A) Huh‐7 cells were treated with the indicated concentrations of BS008 for 24 h and then harvested. Equal amounts of whole‐cell lysates (20 μg) were separated using SDS/PAGE and immunoblotted with various antibodies as indicated. Tubulin is shown as the internal standard. (B, C) Huh‐7 cells were transfected with siRNA‐hnRNP I, siRNA‐snRNP70, or both for 72 h. The protein expression of hnRNP I and snRNP70 was detected by western blot analysis, and the AS of apoptotic gene transcripts was detected by RT‐PCR. (D) mRNA was extracted and detected using RT‐PCR for the AS of various gene transcripts as indicated. The splicing isoforms are illustrated in the bottom row, and their expected PCR products derived through the primers are indicated by arrowheads. M, marker.

Because BS008 could modulate the AS of pre‐mRNA and the computational algorithms predicted that BS008 might influence the splicing factors hnRNP I, snRNP70, and U2AF2, we used western blotting to analyze the expression levels of these three splicing factors. The results showed that the expression levels of hnRNP I and snRNP70 were downregulated, but U2AF2 did not alter significantly after BS008 treatment (Fig. 5A). This suggested that BS008 might modulate AS through decreasing the expression of hnRNP I and snRNP70. Hence, knockdown experiments were performed to investigate the probable modulation mechanism of BS008. Huh‐7 cells were transfected with 20 nm hnRNP I and/or si‐snRNP70 for 72 h, and the efficiency of the reduction of the hnRNP I and snRNP70 was validated by western blot (Fig. 5B). We discovered that hnRNP I knockdown decreased the proportion of *HIPK3 U*(*+*) variants, but it had no significant effect on the AS of *BCL‐X* and *SMAC* transcripts. Furthermore, snRNP70 knockdown increased the proportion of *HIPK3 U*(*+*) and *SMAC‐3* variants, but it had no significant effect on the AS of *BCL‐X* transcripts. The combination of hnRNP I and snRNP70 knockdown increased the proportion of *SMAC‐3* variants, but it had no significant effect on the AS of *BCL‐X* and *HIPK3* transcripts in the Huh‐7 cells (Fig. 5C). The results of the combined knockdown were not entirely consistent with those of BS008 treatment, which suggested that other splicing factors might be involved in BS008‐induced splicing regulation. Therefore, we explored the expression levels of SRSF1, SRSF3, hnRNP A1, hnRNP A2/B1, and hnRNP C1/C2 in BS008 treatment. The results showed that the phosphorylation levels of SRSF1 and SRSF3 were decreased, and the expression levels of hnRNP C1/C2 were downregulated, but hnRNP A1 and hnRNP A2/B1 did not alter significantly after BS008 treatment (Fig. 5A). These results implied that BS008 could modulate AS through changes in the phosphorylation levels or expression levels of various splicing factors. Moreover, the splicing factors have been reported to possibly be subject to AS (Lazar and Goodman, 2000; Robinson and Smith, 2006). Thus, we also evaluated the AS of the splicing‐related gene transcripts that changed at the protein level in BS008 treatment. We found that BS008 increased the proportion of *hnRNP C Delta Ex2* and *hnRNP I Delta Ex2* variants, but it did not significantly affect the AS of *SRSF1*,* SRSF3*, and *snRNP70* transcripts in the Huh‐7 cells (Fig. 5D). Similar splicing patterns were observed in K562 cells and T315I cells (Fig. S3).

### 3.6 Effect of BS008 on AKT kinase, SRPK1, SRPK2, and PP1

**Figure 6:**
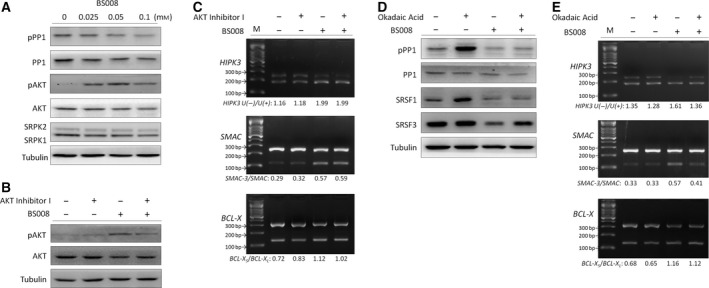
Effect of BS008 on AKT kinase, SRPK1, SRPK2, and PP1. (A) Huh‐7 cells were treated with the indicated concentrations of BS008 for 24 h. The proteins were analyzed using western blot. (B, C) Huh‐7 cells were treated with 10 μm AKT inhibitor for 1 h and then exposed to 0.1 mm BS008 for 24 h. (D, E) Huh‐7 cells were treated with 20 nm OA for 1 h and then exposed to 0.1 mm BS008 for 24 h. The proteins and mRNA were analyzed through western blot and RT‐PCR, respectively. Western blot: Equal amounts of whole‐cell lysates (20 μg) were separated using SDS/PAGE and immunoblotted with various antibodies as indicated. Tubulin is shown as the internal standard. RT‐PCR: mRNA was extracted and detected for the AS of *BCL‐X*,* HIPK3*, and *SMAC* transcripts. M, marker.

During AS, the kinases and phosphatases can be involved in regulating the phosphorylation status of splicing factors (Naro and Sette, 2013). We also explored the effect of BS008 on AKT kinase, PP1, and SRPKs in Huh‐7 cells. The results showed that BS008 reduced the expression levels of SRPK1 and SRPK2, activated AKT kinase by increasing the phosphorylation of AKT at Ser473, and activated PP1 by decreasing the phosphorylation of PP1 at Thr320 (Fig. 6A). To explore the possible role of AKT kinase and PP1 in the modulation of AS in BS008 treatment, the AKT and PP1 inhibitors were used. We pretreated Huh‐7 cells with 10 μm AKT inhibitor for 1 h before treatment with 0.1 mm BS008, and the efficiency of the reduction of the phosphorylation level of AKT (Ser473) was validated by western blot (Fig. 6B). The results showed that AKT inhibitor had no significant effect on the AS of *BCL‐X*,* HIPK3*, and *SMAC* transcripts in the BS008‐treated Huh‐7 cells (Fig. 6C). By contrast, we pretreated Huh‐7 cells with 20 nm PP1 inhibitor okadaic acid (OA) (Cohen *et al*., 1989) for 1 h before treatment with 0.1 mm BS008. Although there was no significant effect of OA on the AS of *BCL‐X* transcripts, OA pretreatment could relieve the effects of BS008 on *HIPK3* and *SMAC* splicing in Huh‐7 (Fig. 6E), as well as effects of BS008 on the dephosphorylation of SRSF3 (Fig. 6D). These results suggested that other splicing factors such as hnRNPs might be involved in the *BCL‐X* splicing process, and PP1 played an important role in modulating phosphorylation of SRSF3 and hence the modulation of *HIPK3* and *SMAC* in Huh‐7 cells after BS008 treatment. Pretreatment of the leukemic K562 cells with OA to inhibit PP1 phosphatase activity abrogated the effects of BS008 on *BCL‐X*,* HIPK3*, and *SMAC* splicing (Fig. S4). Taken together, these results suggested that PP1 phosphatase rather than AKT kinase was associated with the dephosphorylation of SRSF3, which in turn played a role in the BS008‐modified AS process. In this regard, BS008 appears similar to amiloride, which regulates the AS through a PP1‐mediated splicing mechanism (Chang *et al*., 2011a,2011b, 2017).

### 3.7 BS008 affects histone‐tail post‐translational modifications

**Figure 7:**
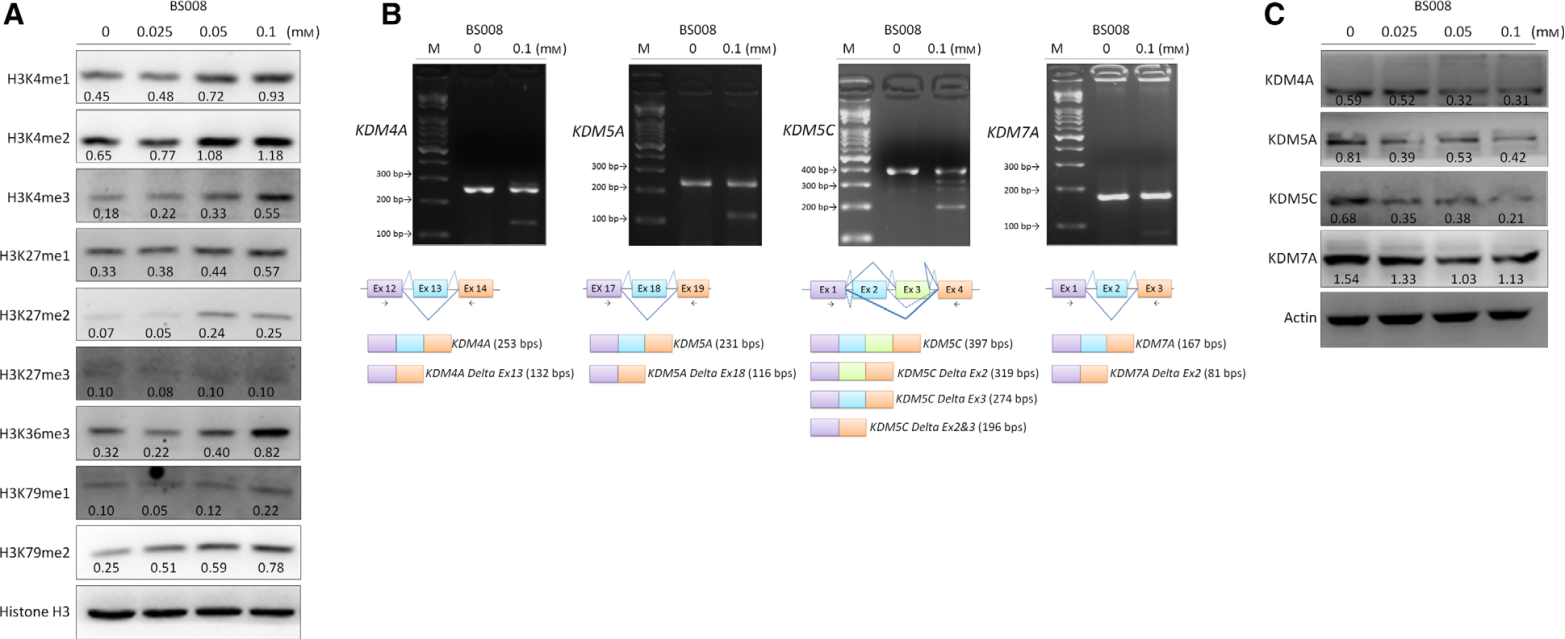
BS008 affects histone modification. Huh‐7 cells were treated with BS008 at the indicated concentrations for 24 h and then harvested. Equal amounts of whole‐cell lysates (20 μg) were separated using SDS/PAGE and immunoblotted with various antibodies as indicated in (A) histone‐tail PTMs and (C) histone modification enzymes. Histone H3 and tubulin are shown as the internal standards. (B) Messenger RNA was extracted and detected using RT‐PCR for the AS of various gene transcripts as indicated. The splicing isoforms are illustrated in the bottom row, and the expected PCR products derived through the primers are indicated by arrowheads. Densitometric values are shown at the bottom of the blot. M, marker.

Several studies have revealed the correlation between histone‐tail post‐translational modifications (PTMs) and AS. Specific histone marks can affect the splicing outcome by recruiting splicing factors to the site of transcription (Podlaha *et al*., 2014; Zhou *et al*., 2014). Moreover, we have demonstrated that amiloride reversed aberrant histone modification patterns, resulting in the disruption of the association of splicing complex with the transcripts (Chang *et al*., 2017). To investigate whether histone‐tail PTMs are involved in the BS008‐regulated splicing, we analyzed the effects of BS008 on histone‐tail PTMs in Huh‐7 cells. The results showed that BS008 increased the expression levels of H3K4me1, H3K4me2, H3K4me3, H3K27me1, H3K27me2, H3K36me3, and H3K79me2, except H3K27me3 and H3K79me1 (Fig. 7A). We further investigated whether BS008 increased the expression levels of histone methylation through affecting the expression of histone‐modifying enzymes. Western blot and PCR analysis confirmed that BS008 reduced the protein levels of histone demethylases, KDM4A, KDM5A, KDM5C, and KDM7A (Fig. 7C), through modulating the AS of these enzymes (Fig. 7B). These results implied that alterations in histone‐tail PTM patterns after BS008 treatment might involve in BS008‐induced splicing regulation.

### 3.8 BS008 affects the genome‐wide AS detected by RNA sequencing

**Figure 8:**
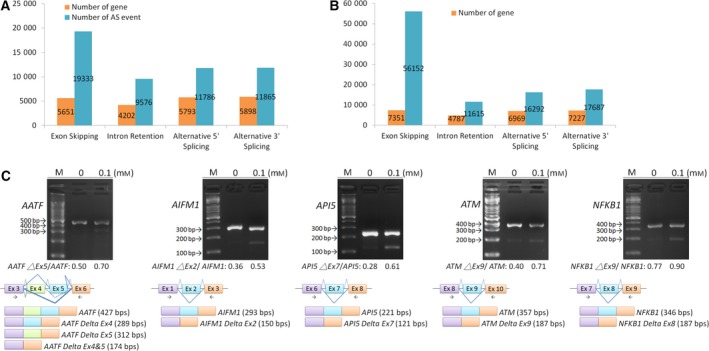
BS008 affects genome‐wide AS detected using RNA sequencing. Huh‐7 cells were treated with (A) 0.1% DMSO and (B) 0.1 mm BS008 for 24 h prior to RNA sequencing. Gray bars indicate the number of AS events, and white bars indicate the number of genes in which AS events occurred. The number shown above the bar is the event. (C) Huh‐7 cells were treated with the indicated concentrations of BS008 for 24 h and then harvested. mRNAs were extracted and detected using RT‐PCR for the AS of various gene transcripts. The splicing isoforms are illustrated in the bottom row, and their expected PCR products derived through the primers are indicated by arrowheads. M, marker.

The ability of BS008 to modulate histone‐tail PTM patterns and the phosphorylation and expression levels of splicing‐related proteins implied that it might have a wide range of effects on the AS of pre‐mRNA. Therefore, we used whole‐RNA sequencing to evaluate global genes in BS008‐treated Huh‐7 cells and found that BS008 could affect the AS of various gene transcripts and the frequency of AS (Fig. 8A,B). Top 500 altered transcripts regulated by BS008 could be classified into functional categories involving pathways in cancer, spliceosome, cell cycle, and apoptosis. We randomly selected five apoptotic genes (*AATF*,* ATM*,* AIFM1*,* NFKB1*, and *API5)* and further validated the changes in AS. As shown in Fig. 8C, the AS patterns of these genes were similar to the results of RNA sequencing in BS008 treatment. These results indicated that BS008 had a genome‐wide effect on the AS of pre‐mRNA.

### 3.9 BS008 arrested the cell cycle at the G_2_/M phase and induced apoptosis

**Figure 9:**
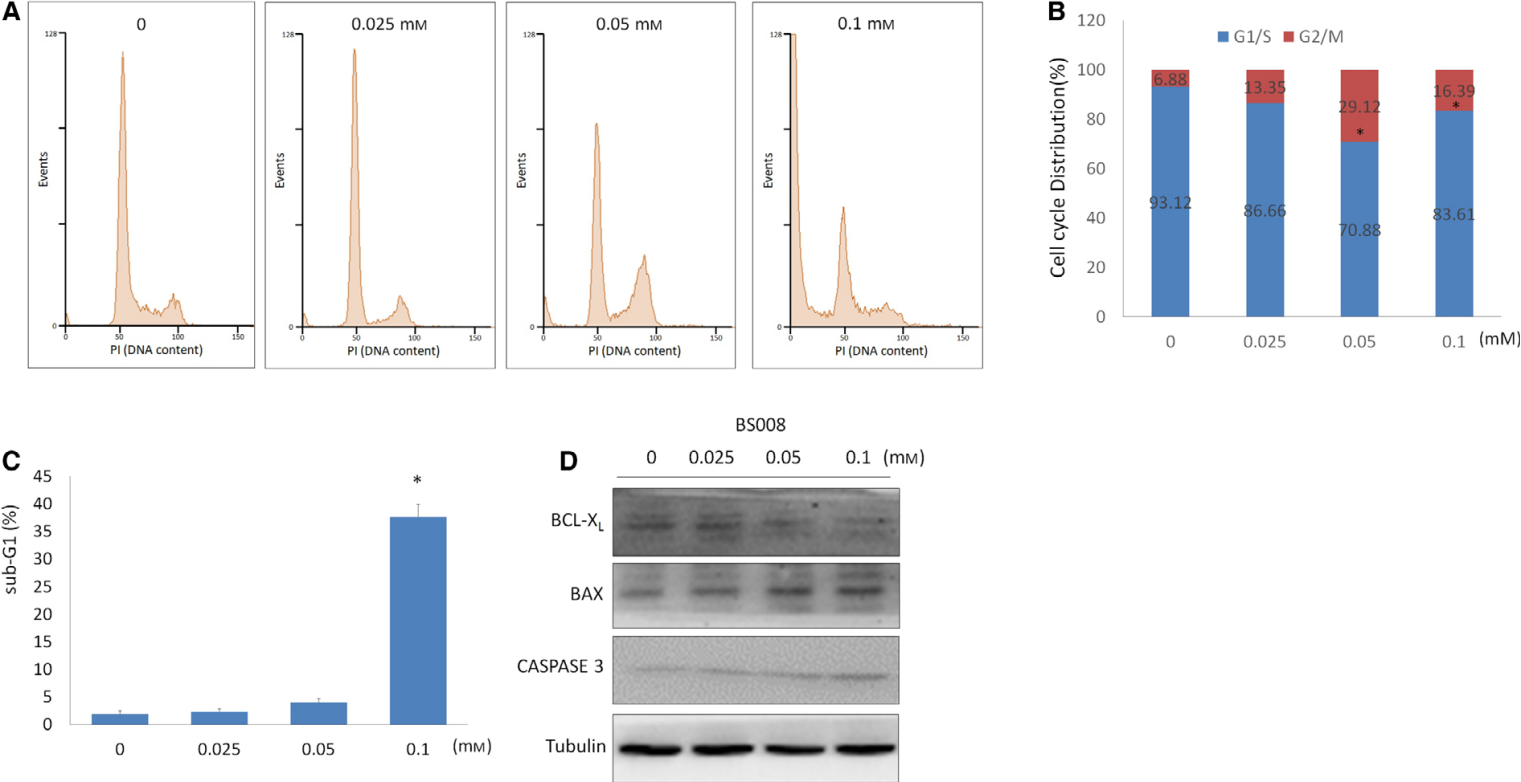
BS008 arrested the cell cycle at the G_2_/M phase and induced apoptosis. Huh‐7 cells were treated with BS008 at the indicated concentrations for 24 h and then stained with PI. (A) DNA content was analyzed using flow cytometry. (B) Histogram of the cell cycle distribution. Student's *t*‐tests (unpaired, two‐tailed) were used to compare the cell cycle G_2_/M distribution between BS008 treatment and control (*P < 0.05). (C) Histogram of the apoptotic sub‐G_1_ population. Data are presented as mean ± SD from three independent experiments (*P < 0.05 using unpaired two‐tailed Student's *t*‐test). (D) Huh‐7 cells were treated with the indicated concentrations of BS008 for 24 h and then harvested. Equal amounts of whole‐cell lysates (20 μg) were separated using SDS/PAGE and immunoblotted with various antibodies as indicated. Tubulin is shown as the internal standard.

Our results showed that the AS of apoptotic genes was affected by BS008 treatment. To further investigate the effects of BS008 on the inhibition of cell growth, we analyzed the effect of BS008 on cell cycle distribution using flow cytometry; the results showed that administration of BS008 could arrest Huh‐7 cells at the G_2_/M phase (Fig. 9A,B) and increase the quantity of apoptotic sub‐G_1_‐phase cells (Fig. 9C). Moreover, western blot results showed that BS008 increased the expression levels of BAX and the active form of CASPASE‐3, while it decreased the expression levels of BCL‐X_L_ (Fig. 9D). These data indicated that BS008 could affect cell cycle progression and induced cell apoptosis.

### 3.10 Inhibitory effect of BS008 on tumor growth in a xenograft animal model

**Figure 10:**
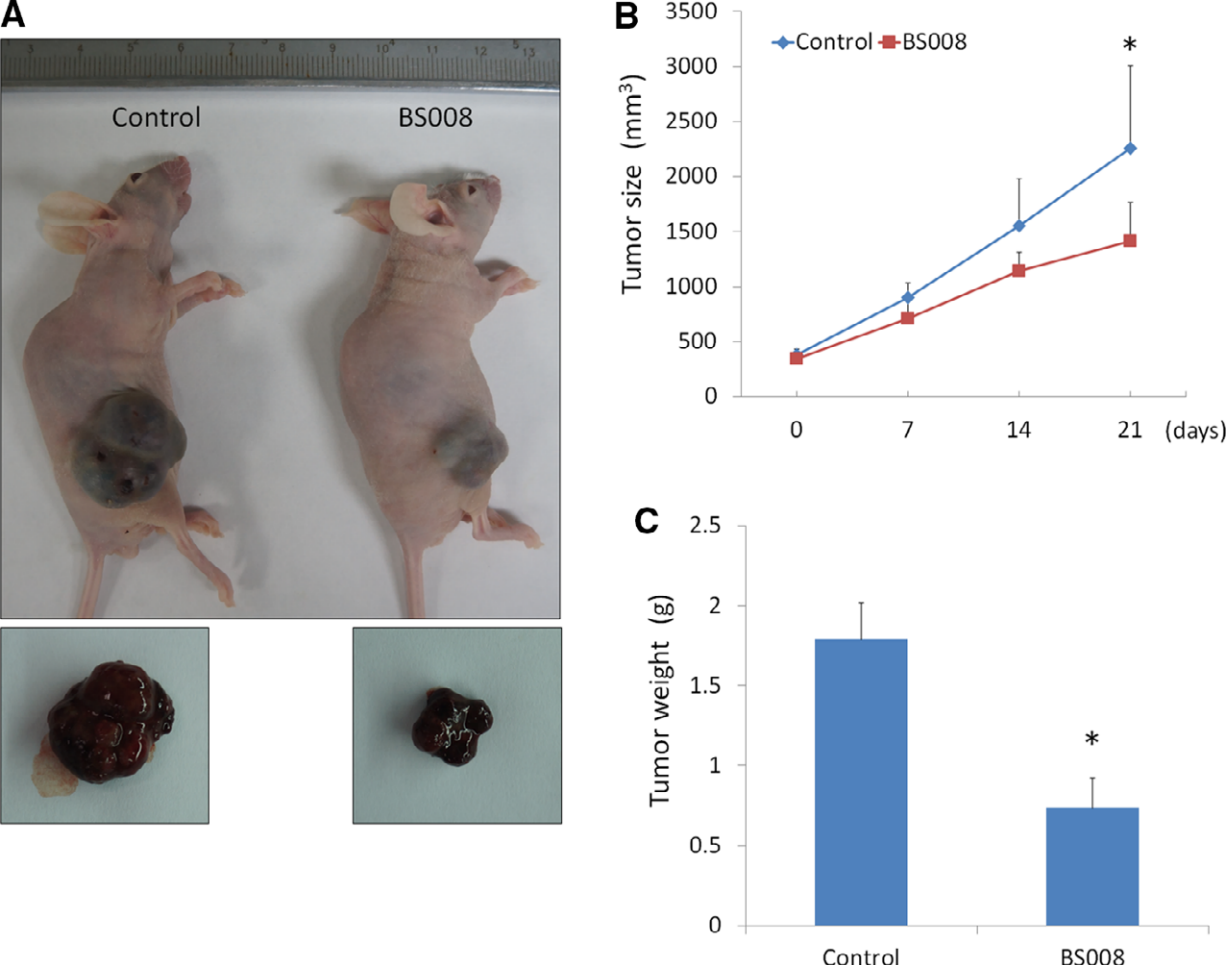
Inhibitory effect of BS008 on tumor growth in a xenograft animal model. Six male nude mice bearing Huh‐7 cell tumors were treated with a solvent (control) or BS008 (30.9 μg/10 g) for 21 days. (A) Representative picture of tumor growth in xenograft nude mice administered solvent (left) and BS008 (right). (B) Tumor volumes were measured after therapy was initiated. (C) Histogram of tumor weight. Data are presented as mean ± SD from six independent experiments (*P < 0.05 using unpaired two‐tailed Student's *t*‐test).

Because we observed the inhibition of cancer cell viability by BS008 *in vitro*, we investigated whether these observations could be translated into an animal model system *in vivo*. Huh‐7 cells were inoculated subcutaneously at the right flank of six male immunodeficient athymic mice. When the tumors reached a size of approximately 300 mm^3^, the mice received subcutaneous injection of BS008 or control solvent every other day, and the sizes of the tumors were monitored every week. After 3 weeks of treatment, tumor growth was significantly suppressed under the influence of BS008 treatment. Representative picture of tumor growth in xenograft nude mice administered solvent (left) and BS008 (right) is shown in Fig. 10A. The six average tumor sizes in the BS008‐treated and control groups were 1484.87 and 2362.8 mm^3^, respectively (Fig. 10B). At the end of treatment, the tumor tissue was isolated and weighed. The tissue extracted from the BS008‐treated group weighed considerably less than that extracted from the control group (0.73 vs. 1.79 g; Fig. 10C). These findings demonstrated that BS008 administration significantly inhibited Huh‐7 xenograft growth *in vivo*.

### 3.11 BS008 potentiates the growth‐inhibitory effect of sorafenib and nilotinib

**Figure 11:**
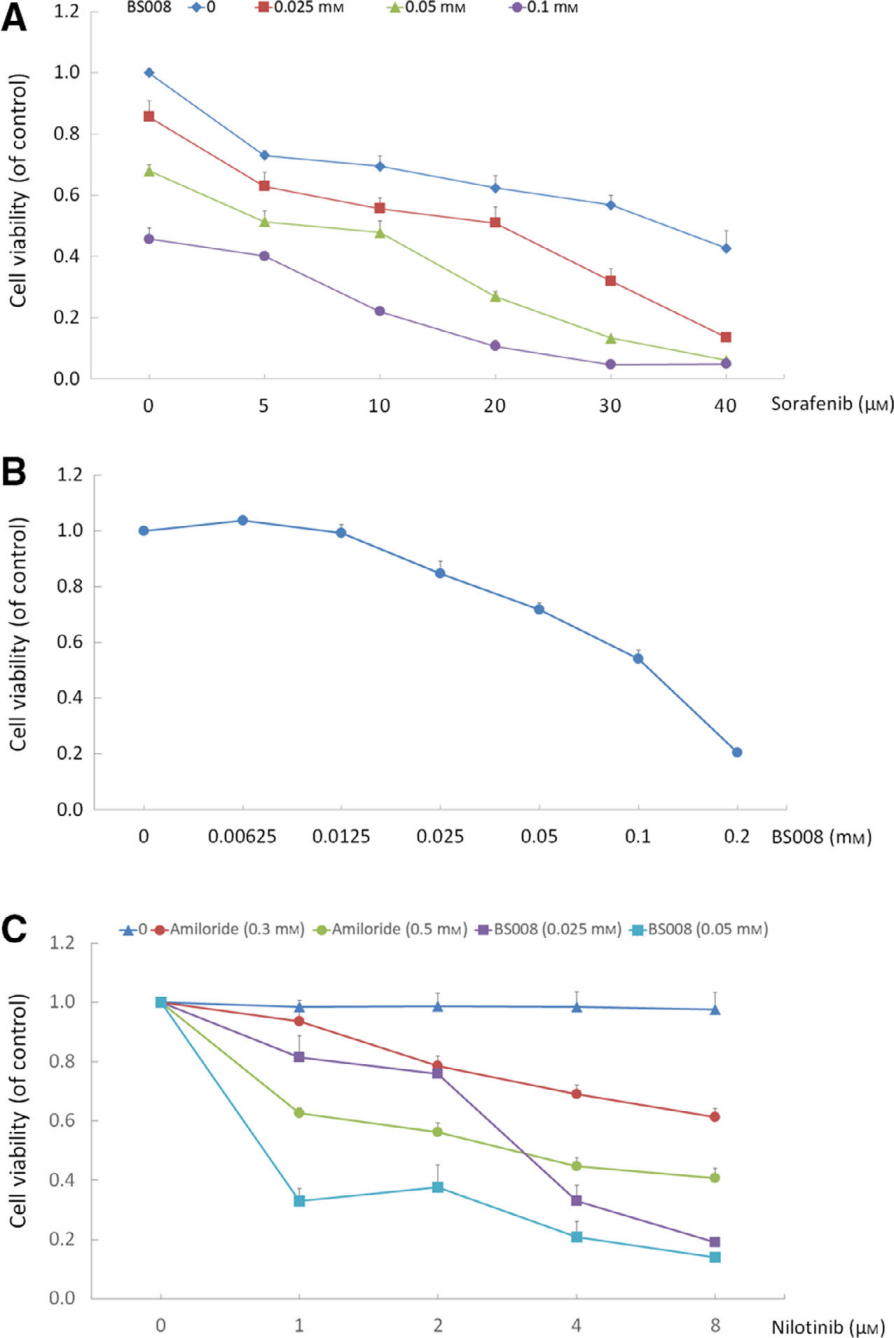
Treatment with BS008 enhances the sensitivity of cancer cells to sorafenib or nilotinib. (A) Huh‐7 cells were treated with BS008, sorafenib, or both at the indicated concentrations for 24 h. (B) T315I cells were treated with BS008 at the indicated concentrations for 24 h. (C) T315I cells were treated with a constant concentration of BS008 or amiloride in combination with nilotinib for 24 h. MTT assays were used to assess cell viability. Data are presented as mean ± SD from three independent experiments.

Pharmaceutical combinations have been widely used in clinical applications, displaying advantages over the use of individual product (Liu *et al*., 2014). Currently, sorafenib is the only first‐line treatment approved for HCC by the US Food and Drug Administration, but the complete response rate to sorafenib in HCC is relatively low (0.7–3%) (Arao *et al*., 2013). To investigate if BS008 can potentiate the growth‐inhibitory effect of sorafenib, Huh‐7 cells were treated with BS008, sorafenib, or both. We found BS008 and sorafenib alone caused a dose‐dependent loss of cell viability. A combination of BS008 at a concentration of 0.025 mm and sorafenib at a concentration of 20, 30, and 40 μm inhibited Huh‐7 viability to 0.52, 0.32, and 0.14, respectively (Fig. 11A). These data indicated that combination of BS008 and sorafenib produced synergistic effects. As amiloride exhibited the synergistic effect in combination with cytotoxic chemotherapeutic agents on T315I cells (Chang *et al*., 2011b), we further investigated whether BS008 exerted a therapeutically beneficial effect when administered in combination with nilotinib. As shown in Fig. 11B,C, BS008 exhibited inhibitory effect alone or in a synergistic combination with nilotinib. Notably, BS008 showed a much more potent effect than amiloride when combined with nilotinib (Fig. 11C).

## 4 Discussion

The phosphorylation state of splicing factors has been reported to affect almost all steps of AS (Misteli, 1999). In general, AKT can directly phosphorylate SR proteins (Bavelloni *et al*., 2014; Shultz *et al*., 2010) or indirectly act on splicing regulation by SRPKs, leading to the translocation of SRPKs into the nucleus and thereby modifying SR protein activities (Zhou *et al*., 2012). Thus, BS008 decreasing the expression levels of SRPKs may cause AKT to fail to phosphorylate SR proteins through the AKT–SRPK–SR pathway. However, increased phosphorylation levels of AKT were also found in BS008‐treated cells, which may be a compensatory activation that attempts to ‘normalize’ the phosphorylation state of SR proteins. By contrast, pretreatment with the PP1 inhibitor OA could partially restore the effects of BS008 on the phosphorylation levels of SRSF3 as well as the AS of *HIPK3* and *SMAC* transcripts. These results are similar to those of other reports that revealed that 4β‐hydroxywithanolide E (Lee *et al*., 2017), ceramide (Chalfant *et al*., 2002; Massiello *et al*., 2004), emetine (Boon‐Unge *et al*., 2007; Pan *et al*., 2011), and epigallocatechin‐3‐gallate/ibuprofen can modulate (Kim, 2008) the AS of apoptotic gene transcripts through a PP1‐mediated splicing mechanism. These publications imply that PP1 plays a crucial role in regulating the AS of apoptotic gene transcripts. However, we found PP1 mediated the effects of BS008 on the AS through the dephosphorylation of SRSF3 proteins, and it mediated the effects of amiloride through the dephosphorylation of SRSF1 proteins. This might be the different functional group of amiloride derivatives, making the interaction between the compounds and proteins differ and leading to diverse regulation of AS. The results showed that BS008 could regulate the AS of *hnRNP C*,* hnRNP I*,* KDM4A*,* KDM5A*,* KDM5C*, and *KDM7A* transcripts. The lost exon of *hnRNP* C *Delta Ex2* will cause the loss of the start codon, and the lost exon of *hnRNP I Delta Ex2*,* KDM4A Delta Ex13*,* KDM5A Delta Ex18*,* KDM5C Delta Ex2* and *Delta Ex2&3*, and *KDM7A Delta Ex2* will cause frameshifts, which may contribute to the decline of protein levels in BS008‐treated cells. Changes in the expression levels of KDM4A, KDM5A, KDM5C, and KDM7A are correlated with alterations in H3K4me1, H3K4me2, H3K4me3, H3K27me1, H3K27me2, and H3K36me3 levels (Kooistra and Helin, 2012) as well as the transcripts of their target RNA. For example, KDM5B is enriched nearby alternative exons, and the reduction of KDM5B causes altered levels of H3K4 methylation in alternative splice exon regions (He and Kidder, 2017); furthermore, knocking down KDM4A increases H3K36me3 levels and increases intron retention (Sen *et al*., 2017). BS008 could alter the AS of the genes of splicing factors and histone modification enzymes, and the proteins translated by these genes affect the regulation of AS. Their causal or feedback mechanism needs to be further explored.

In addition to the effects of the AS of *BCL‐X*,* HIPK3*, and *SMAC* transcripts, the results of RNA sequencing showed that other apoptotic genes such as *AATF*,* AIFM1*, and *ATM* could be regulated by BS008. The transcriptional cofactor AATF is an RNA polymerase II and a central regulator of the p53‐driven DNA damage response. Evidence has indicated that increased expression levels of AATF were found in various cancerous tissues and are negatively correlated with patient survival in neuroblastomas (Fanciulli *et al*., 2000; Hopker *et al*., 2012). Moreover, studies have shown that the loss of AATF inhibits proliferation and promotes apoptosis in MG‐63 cells by decreasing the level of mutant p53 (Liu *et al*., 2016). The present study showed that an increased proportion of *AATF Delta Ex5* was found in BS008‐treated cells. The lost exon of *AATF Delta Ex5* led to a frameshift and a premature stop at codon 298, which suggested that the deviated AATF that was translated from *AATF Delta Ex5* might be involved in BS008‐induced apoptosis. AIFM1 is found in the inner mitochondrial membrane and has a dual role as an NADH oxidoreductase and regulator of apoptosis. A defect in AIFM1 can lead to a loss of prosurvival function associated with the assembly/stabilization of the mitochondrial electron transfer chain, resulting in promotion of the translocation of AIFM1 from mitochondria to the nucleus, and finally causing large‐scale DNA cleavage (Ardissone *et al*., 2015; Polster, 2013). Cell‐free systems also showed that AIFM1 could cause mitochondrial membrane permeabilization and trigger the release of cytochrome c, indicating that AIFM1 may be involved in a positive feedback loop of the apoptosis pathway (Susin *et al*., 1999). Our results showed that the proportion of *AIFM1 Delta Ex2* was increased in BS008‐treated cells, and the lost exon 2 of *AIFM1* caused a frameshift, wherein the shifted frame did not encounter a new stop codon. This implied that the AIFM1 deficiency of BS008‐treated cells may result in the loss of respiratory elements and, therefore, impaired mitochondrial oxidative phosphorylation, eventually leading to apoptosis. ATM is a serine/threonine protein kinase that can be recruited and activated by DNA double‐strand breaks (DSBs) or genotoxic stress, thereby acting as a DNA damage sensor. In response to DNA damage, ATM phosphorylates various downstream proteins, such as CHK2, histone H2AX, and TP53, to start a complex signaling cascade, and it then induces cell cycle arrest, increases DNA repair, and inhibits apoptosis (Hollingworth and Grand, 2015; Spriggs and Laimins, 2017). Studies have also indicated that ATM‐deficient cells derived from ataxia–telangiectasia patients are distinctly sensitive to radiation and defective in DSB repair (Marechal and Zou, 2013). We found that BS008 increased the proportion of *ATM Delta Ex9,* which is able to cause a frameshift and a premature stop at codon 372. Thus, this improper ATM may not only have severe consequences in repairing DNA damage, but also affect cell cycle distribution and apoptosis in BS008‐treated cells. Based on these findings, BS008 probably has an effect on apoptosis signaling pathways through regulating the AS of various apoptosis‐related gene transcripts, making it potentially useful in cancer treatment.

Sorafenib is a multikinase inhibitor that represses tumor‐cell proliferation and angiogenesis and promotes tumor‐cell apoptosis by inhibiting the Raf/MEK/ERK signaling pathway and VEGF receptor tyrosine kinase. Although sorafenib is the standard first‐line systemic drug for HCC, primary and acquired resistance to sorafenib results in limited benefits. Primary resistance of HCC to sorafenib is caused by genetic heterogeneity and may be associated with overexpression of EGFR and abnormal activation of its downstream molecules Ras/Raf/MEK/ERK. Acquired resistance is possibly related to activation of the compensatory pathways, such as cross talk involving JAK–STAT pathways (Zhu *et al*., 2017). In this study, the results of RNA sequencing showed that BS008 affected not only the AS of *EGFR* and *ERK* transcripts, but also the AS of *STAT3*,* Mcl‐1,* and *cyclin D1* transcripts (data not shown). This suggested that BS008 might potentiate the growth‐inhibitory effect of sorafenib through modulation of these gene transcripts. However, detailed examination is still required to explore sorafenib resistance‐related genes in BS008‐induced AS.

## Conclusions

**Figure 12:**
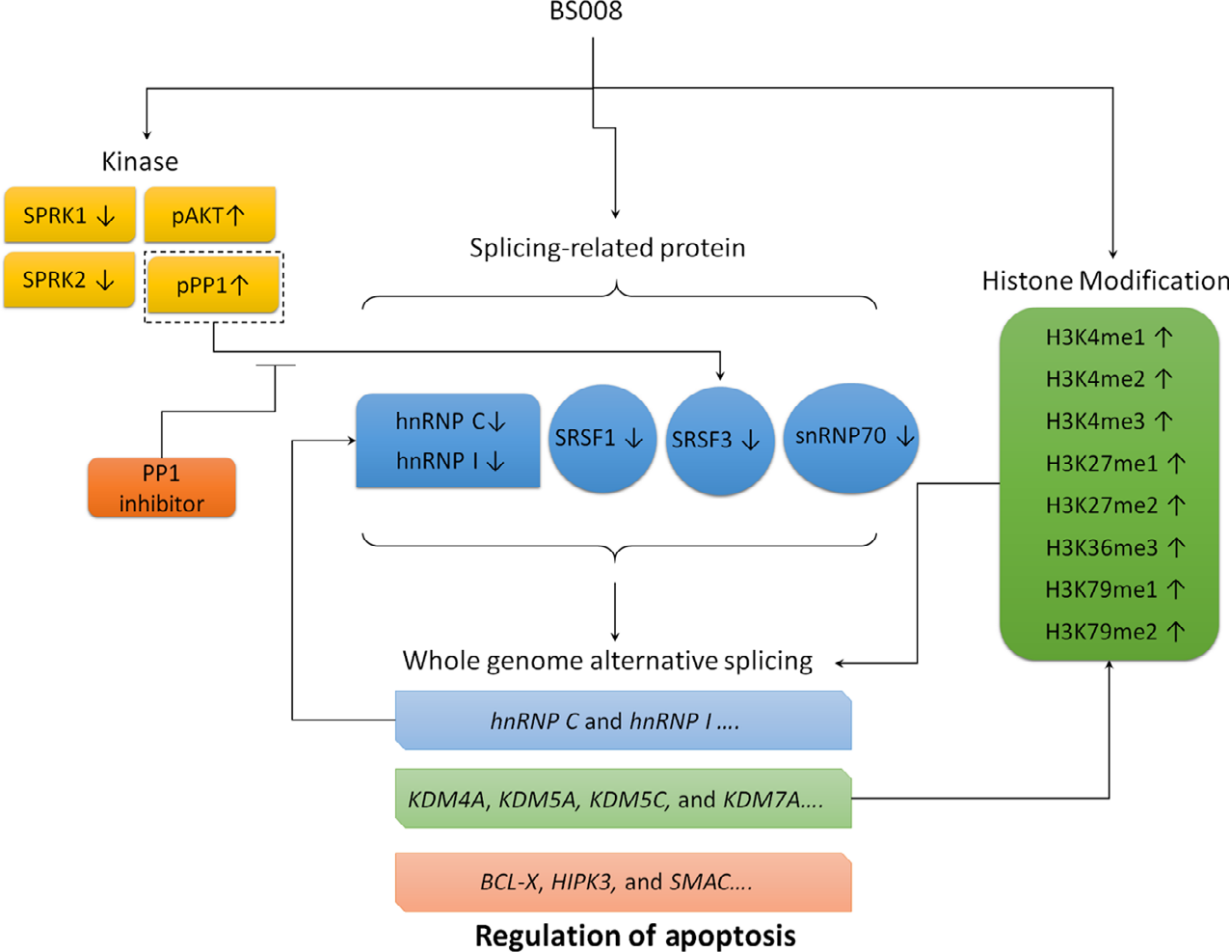
Hypothetical schematic of BS008‐induced AS in Huh‐7 cells.

We found that BS008, an amiloride derivative, is more effective than amiloride in cancer treatment. BS008 has an effect not only on histone‐tail PTMs but also on the expression and phosphorylation state of splicing factors, resulting in the genome‐wide effects on the AS of gene transcripts (Fig. 12). In addition, BS008 can sensitize cancer cells to sorafenib and nilotinib, indicating that a combination of BS008 and sorafenib or nilotinib is a promising therapeutic strategy for clinical application in the treatment of cancer.

## Conflict of interest

The authors declare no conflict of interest.

## Author contributions

C‐CL and W‐HC contributed equally to this manuscript. C‐CL, W‐HC, S‐YL, Y‐CW, and J‐GC designed the study. C‐CL, W‐HC, Y‐SC, J‐MY, C‐SC, K‐CH, Y‐TC, T‐YL, and Y‐CC acquired and analyzed the data. J‐MY, S‐YL, J‐GC, and Y‐CW interpreted the data. C‐CL, W‐HC, and J‐GC drafted and critically revised the manuscript.

## Supporting information


**Fig. S1**. Effects of BS008 on alternative splicing of (A) BCL‐X, (B) HIPK3, and (C) SMAC RNAs. Messenger RNA was extracted and detected using RT‐PCR for the AS in K562, T315I and normal mononuclear cells.

**Fig. S2**. Effects of HIPK3 splicing on BS008‐induced cell death. (A) Experimental design and vectors. (B) K562 cells were transfected with the expression plasmids as indicated for 24 h prior to BS008 treatment. RT‐PCR validation of endogenous HIPK3 alternative splicing (upper panel). Cell viability was analyzed using MTT assay followed by 24‐h treatment with BS008 (lower panel). Data are presented as mean ± standard deviation from six independent experiments (*P < 0.05 using unpaired two‐tailed Student's *t*‐test).

**Fig. S3**. Effects of BS008 on alternative splicing of (A) snRNP70, (B) SRSF1, (C) SRSF3, (D) hnRNP C, and (E) hnRNP I RNAs. Cells were treated with BS008 for 24 h and then harvested for RT‐PCR analysis.

**Fig. S4**. Effects of PP1 phosphatase on BS008‐induced alternative splicing. RT‐PCR results from K562 cells pretreated with (+) or without (‐) okadaic acid and then exposed to BS008 for 24 h.

**Table S1.** Information about the Small interfering RNA used in the study.
**Table S2.** Information about the primers used in reverse transcription polymerase chain reaction.
**Table S3.** Ten known target proteins of amiloride collected from BindingDB.
**Table S4.** Alternative splicing‐related proteins.

## References

[mol212524-bib-0001] Agrawal AA , McLaughlin KJ , Jenkins JL and Kielkopf CL (2014) Structure‐guided U2AF65 variant improves recognition and splicing of a defective pre‐mRNA. Proc Natl Acad Sci USA 111, 17420–17425. 25422459 10.1073/pnas.1412743111 PMC4267390

[mol212524-bib-0002] Arao T , Ueshima K , Matsumoto K , Nagai T , Kimura H , Hagiwara S , Sakurai T , Haji S , Kanazawa A , Hidaka H *et al* (2013) FGF3/FGF4 amplification and multiple lung metastases in responders to sorafenib in hepatocellular carcinoma. Hepatology 57, 1407–1415. 22890726 10.1002/hep.25956

[mol212524-bib-0003] Ardissone A , Piscosquito G , Legati A , Langella T , Lamantea E , Garavaglia B , Salsano E , Farina L , Moroni I , Pareyson D *et al* (2015) A slowly progressive mitochondrial encephalomyopathy widens the spectrum of AIFM1 disorders. Neurology 84, 2193–2195. 25934856 10.1212/WNL.0000000000001613 PMC4451043

[mol212524-bib-0004] Bavelloni A , Piazzi M , Faenza I , Raffini M , D'Angelo A , Cattini L , Cocco L and Blalock WL (2014) Prohibitin 2 represents a novel nuclear AKT substrate during all‐trans retinoic acid‐induced differentiation of acute promyelocytic leukemia cells. Faseb j 28, 2009–2019. 24522204 10.1096/fj.13-244368

[mol212524-bib-0005] Boon‐Unge K , Yu Q , Zou T , Zhou A , Govitrapong P and Zhou J (2007) Emetine regulates the alternative splicing of Bcl‐x through a protein phosphatase 1‐dependent mechanism. Chem Biol 14, 1386–1392. 18096507 10.1016/j.chembiol.2007.11.004 PMC2186211

[mol212524-bib-0006] Bull MB and Laragh JH (1968) Amiloride. A potassium‐sparing natriuretic agent. Circulation 37, 45–53. 5634728 10.1161/01.cir.37.1.45

[mol212524-bib-0007] Chalfant CE , Rathman K , Pinkerman RL , Wood RE , Obeid LM , Ogretmen B and Hannun YA (2002) *De novo* ceramide regulates the alternative splicing of caspase 9 and Bcl‐x in A549 lung adenocarcinoma cells. Dependence on protein phosphatase‐1. J Biol Chem 277, 12587–12595. 11801602 10.1074/jbc.M112010200

[mol212524-bib-0008] Chang WH , Liu TC , Yang WK , Lee CC , Lin YH , Chen TY and Chang JG (2011b) Amiloride modulates alternative splicing in leukemic cells and resensitizes Bcr‐AblT315I mutant cells to imatinib. Cancer Res 71, 383–392. 21224352 10.1158/0008-5472.CAN-10-1037

[mol212524-bib-0009] Chang WH , Niu DM , Lu CY , Lin SY , Liu TC and Chang JG (2017) Modulation the alternative splicing of GLA (IVS4 + 919G>A) in Fabry disease. PLoS ONE 12, e0175929. 28430823 10.1371/journal.pone.0175929 PMC5400244

[mol212524-bib-0010] Chang JG , Yang DM , Chang WH , Chow LP , Chan WL , Lin HH , Huang HD , Chang YS , Hung CH and Yang WK (2011a) Small molecule amiloride modulates oncogenic RNA alternative splicing to devitalize human cancer cells. PLoS ONE 6, e18643. 21694768 10.1371/journal.pone.0018643 PMC3111415

[mol212524-bib-0011] Chen YF , Hsu KC , Lin SR , Wang WC , Huang YC and Yang JM (2010) SiMMap: a web server for inferring site‐moiety map to recognize interaction preferences between protein pockets and compound moieties. Nucleic Acids Res 38, W424–W430. 20519201 10.1093/nar/gkq480 PMC2896162

[mol212524-bib-0012] Chin KH , Lee YC , Tu ZL , Chen CH , Tseng YH , Yang JM , Ryan RP , McCarthy Y , Dow JM , Wang AH *et al* (2010) The cAMP receptor‐like protein CLP is a novel c‐di‐GMP receptor linking cell‐cell signaling to virulence gene expression in *Xanthomonas campestris* . J Mol Biol 396, 646–662. 20004667 10.1016/j.jmb.2009.11.076

[mol212524-bib-0013] Chiu YY , Tseng JH , Liu KH , Lin CT , Hsu KC and Yang JM (2014) Homopharma: a new concept for exploring the molecular binding mechanisms and drug repurposing. BMC Genom 15(Suppl 9), S8. 10.1186/1471-2164-15-S9-S8 PMC4290623 25521038

[mol212524-bib-0014] Cohen P , Klumpp S and Schelling DL (1989) An improved procedure for identifying and quantitating protein phosphatases in mammalian tissues. FEBS Lett 250, 596–600. 2546812 10.1016/0014-5793(89)80803-8

[mol212524-bib-0015] Ewing TJ , Makino S , Skillman AG and Kuntz ID (2001) DOCK 4.0: search strategies for automated molecular docking of flexible molecule databases. J Comput Aided Mol Des 15, 411–428. 11394736 10.1023/a:1011115820450

[mol212524-bib-0016] Fanciulli M , Bruno T , Di Padova M , De Angelis R , Iezzi S , Iacobini C , Floridi A and Passananti C (2000) Identification of a novel partner of RNA polymerase II subunit 11, Che‐1, which interacts with and affects the growth suppression function of Rb. Faseb j 14, 904–912. 10783144 10.1096/fasebj.14.7.904

[mol212524-bib-0017] Fu XD and Ares M Jr (2014) Context‐dependent control of alternative splicing by RNA‐binding proteins. Nat Rev Genet 15, 689–701. 25112293 10.1038/nrg3778 PMC4440546

[mol212524-bib-0018] Gilson MK , Liu T , Baitaluk M , Nicola G , Hwang L and Chong J (2016) BindingDB in 2015: a public database for medicinal chemistry, computational chemistry and systems pharmacology. Nucleic Acids Res 44, D1045–D1053. 26481362 10.1093/nar/gkv1072 PMC4702793

[mol212524-bib-0019] Guda C , Lu S , Scheeff ED , Bourne PE and Shindyalov IN (2004) CE‐MC: a multiple protein structure alignment server. Nucleic Acids Res 32, W100–W103. 15215359 10.1093/nar/gkh464 PMC441602

[mol212524-bib-0020] Hahn CN , Venugopal P , Scott HS and Hiwase DK (2015) Splice factor mutations and alternative splicing as drivers of hematopoietic malignancy. Immunol Rev 263, 257–278. 25510282 10.1111/imr.12241

[mol212524-bib-0021] He R and Kidder BL (2017) H3K4 demethylase KDM5B regulates global dynamics of transcription elongation and alternative splicing in embryonic stem cells. Nucleic Acids Res 45, 6427–6441. 28402433 10.1093/nar/gkx251 PMC5499819

[mol212524-bib-0022] Hollingworth R and Grand RJ (2015) Correction: Hollingworth, R.; Grand, R.J. Modulation of DNA damage and repair pathways by human tumour viruses. Viruses 2015, 7, 2542‐2591. Viruses 7, 3201–3203. 26008701 10.3390/v7052542 PMC4452920

[mol212524-bib-0023] Hopker K , Hagmann H , Khurshid S , Chen S , Hasskamp P , Seeger‐Nukpezah T , Schilberg K , Heukamp L , Lamkemeyer T , Sos ML *et al* (2012) AATF/Che‐1 acts as a phosphorylation‐dependent molecular modulator to repress p53‐driven apoptosis. EMBO J 31, 3961–3975. 22909821 10.1038/emboj.2012.236 PMC3474921

[mol212524-bib-0024] Hsu KC , Cheng WC , Chen YF , Wang WC and Yang JM (2013) Pathway‐based screening strategy for multitarget inhibitors of diverse proteins in metabolic pathways. PLoS Comput Biol 9, e1003127. 23861662 10.1371/journal.pcbi.1003127 PMC3701698

[mol212524-bib-0025] Hsu KC , Sung TY , Lin CT , Chiu YY , Hsu JT , Hung HC , Sun CM , Barve I , Chen WL , Huang WC *et al* (2015) Anchor‐based classification and type‐C inhibitors for tyrosine kinases. Sci Rep 5, 10938. 26077136 10.1038/srep10938 PMC4468516

[mol212524-bib-0026] Kim MH (2008) Protein phosphatase 1 activation and alternative splicing of Bcl‐X and Mcl‐1 by EGCG + ibuprofen. J Cell Biochem 104, 1491–1499. 18348186 10.1002/jcb.21725

[mol212524-bib-0027] Kondo Y , Oubridge C , van Roon AM and Nagai K (2015) Crystal structure of human U1 snRNP, a small nuclear ribonucleoprotein particle, reveals the mechanism of 5’ splice site recognition. Elife, 4, e04986-5004. 10.7554/eLife.04986 PMC4383343 25555158

[mol212524-bib-0028] Kooistra SM and Helin K (2012) Molecular mechanisms and potential functions of histone demethylases. Nat Rev Mol Cell Biol 13, 297–311. 22473470 10.1038/nrm3327

[mol212524-bib-0029] Kramer B , Rarey M and Lengauer T (1999) Evaluation of the FLEXX incremental construction algorithm for protein‐ligand docking. Proteins 37, 228–241. 10584068 10.1002/(sici)1097-0134(19991101)37:2<228::aid-prot8>3.0.co;2-8

[mol212524-bib-0030] Lazar G and Goodman HM (2000) The Arabidopsis splicing factor SR1 is regulated by alternative splicing. Plant Mol Biol 42, 571–581. 10809003 10.1023/a:1006394207479

[mol212524-bib-0031] Lee CC , Chang WH , Chang YS , Liu TY , Chen YC , Wu YC and Chang JG (2017) 4beta‐Hydroxywithanolide E modulates alternative splicing of apoptotic genes in human hepatocellular carcinoma Huh‐7 Cells. Sci Rep 7, 7290. 28779122 10.1038/s41598-017-07472-6 PMC5544667

[mol212524-bib-0032] Lin JC (2017) Therapeutic applications of targeted alternative splicing to cancer treatment. Int J Mol Sci 19, 75–85. 10.3390/ijms19010075 PMC5796025 29283381

[mol212524-bib-0033] Liu M , Wang D and Li N (2016) Che‐1 gene silencing induces osteosarcoma cell apoptosis by inhibiting mutant p53 expression. Biochem Biophys Res Commun 473, 168–173. 27012205 10.1016/j.bbrc.2016.03.073

[mol212524-bib-0034] Liu Y , Wei Q , Yu G , Gai W , Li Y and Chen X (2014) DCDB 2.0: a major update of the drug combination database. Database 2014, 1–6. bau124. 10.1093/database/bau124 PMC4275564 25539768

[mol212524-bib-0035] Loffing J and Kaissling B (2003) Sodium and calcium transport pathways along the mammalian distal nephron: from rabbit to human. Am J Physiol Renal Physiol 284, F628–F643. 12620920 10.1152/ajprenal.00217.2002

[mol212524-bib-0036] Marechal A and Zou L (2013) DNA damage sensing by the ATM and ATR kinases. Cold Spring Harb Perspect Biol 5, 9–25. 10.1101/cshperspect.a012716 PMC3753707 24003211

[mol212524-bib-0037] Massiello A , Salas A , Pinkerman RL , Roddy P , Roesser JR and Chalfant CE (2004) Identification of two RNA cis‐elements that function to regulate the 5’ splice site selection of Bcl‐x pre‐mRNA in response to ceramide. J Biol Chem 279, 15799–15804. 14734550 10.1074/jbc.M313950200

[mol212524-bib-0038] Misteli T (1999) RNA splicing: What has phosphorylation got to do with it? Curr Biol 9, R198–R200. 10209090 10.1016/s0960-9822(99)80128-6

[mol212524-bib-0039] Naro C and Sette C (2013) Phosphorylation‐mediated regulation of alternative splicing in cancer. Int J Cell Biol 2013, 151839. 24069033 10.1155/2013/151839 PMC3771450

[mol212524-bib-0040] Oberstrass FC , Auweter SD , Erat M , Hargous Y , Henning A , Wenter P , Reymond L , Amir‐Ahmady B , Pitsch S , Black DL *et al* (2005) Structure of PTB bound to RNA: specific binding and implications for splicing regulation. Science 309, 2054–2057. 16179478 10.1126/science.1114066

[mol212524-bib-0041] O'Hare T , Walters DK , Stoffregen EP , Jia T , Manley PW , Mestan J , Cowan‐Jacob SW , Lee FY , Heinrich MC , Deininger MW *et al* (2005) *In vitro* activity of Bcr‐Abl inhibitors AMN107 and BMS‐354825 against clinically relevant imatinib‐resistant Abl kinase domain mutants. Cancer Res 65, 4500–4505. 15930265 10.1158/0008-5472.CAN-05-0259

[mol212524-bib-0042] Pan D , Boon‐Unge K , Govitrapong P and Zhou J (2011) Emetine regulates the alternative splicing of caspase 9 in tumor cells. Oncol Lett 2, 1309–1312. 22848307 10.3892/ol.2011.395 PMC3406502

[mol212524-bib-0043] Podlaha O , De S , Gonen M and Michor F (2014) Histone modifications are associated with transcript isoform diversity in normal and cancer cells. PLoS Comput Biol 10, e1003611. 24901363 10.1371/journal.pcbi.1003611 PMC4046914

[mol212524-bib-0044] Polster BM (2013) AIF, reactive oxygen species, and neurodegeneration: a “complex” problem. Neurochem Int 62, 695–702. 23246553 10.1016/j.neuint.2012.12.002 PMC3610861

[mol212524-bib-0045] Robinson F and Smith CW (2006) A splicing repressor domain in polypyrimidine tract‐binding protein. J Biol Chem 281, 800–806. 16282332 10.1074/jbc.M510578200

[mol212524-bib-0046] Sen A , Gurdziel K , Liu J , Qu W , Nuga OO , Burl RB , Huttemann M , Pique‐Regi R and Ruden DM (2017) Smooth, an hnRNP‐L homolog, might decrease mitochondrial metabolism by post‐transcriptional regulation of isocitrate dehydrogenase (Idh) and other metabolic genes in the sub‐acute phase of traumatic brain injury. Front Genet 8, 175. 29187863 10.3389/fgene.2017.00175 PMC5694756

[mol212524-bib-0047] Shultz JC , Goehe RW , Wijesinghe DS , Murudkar C , Hawkins AJ , Shay JW , Minna JD and Chalfant CE (2010) Alternative splicing of caspase 9 is modulated by the phosphoinositide 3‐kinase/Akt pathway via phosphorylation of SRp30a. Cancer Res 70, 9185–9196. 21045158 10.1158/0008-5472.CAN-10-1545 PMC3059118

[mol212524-bib-0048] Spriggs CC and Laimins LA (2017) Human papillomavirus and the DNA damage response: exploiting host repair pathways for viral replication. Viruses 9, 232–245. 10.3390/v9080232 PMC5580489 28820495

[mol212524-bib-0049] Susin SA , Lorenzo HK , Zamzami N , Marzo I , Snow BE , Brothers GM , Mangion J , Jacotot E , Costantini P , Loeffler M *et al* (1999) Molecular characterization of mitochondrial apoptosis‐inducing factor. Nature 397, 441–446. 9989411 10.1038/17135

[mol212524-bib-0050] Sveen A , Kilpinen S , Ruusulehto A , Lothe RA and Skotheim RI (2016) Aberrant RNA splicing in cancer; expression changes and driver mutations of splicing factor genes. Oncogene 35, 2413–2427. 26300000 10.1038/onc.2015.318

[mol212524-bib-0051] Tazi J , Bakkour N and Stamm S (2009) Alternative splicing and disease. Biochim Biophys Acta 1792, 14–26. 18992329 10.1016/j.bbadis.2008.09.017 PMC5632948

[mol212524-bib-0052] Venables JP (2004) Aberrant and alternative splicing in cancer. Cancer Res 64, 7647–7654. 15520162 10.1158/0008-5472.CAN-04-1910

[mol212524-bib-0053] Yang JM and Chen CC (2004) GEMDOCK: a generic evolutionary method for molecular docking. Proteins 55, 288–304. 15048822 10.1002/prot.20035

[mol212524-bib-0054] Yang JM , Chen YF , Tu YY , Yen KR and Yang YL (2007) Combinatorial computational approaches to identify tetracycline derivatives as flavivirus inhibitors. PLoS ONE 2, e428. 17502914 10.1371/journal.pone.0000428 PMC1855430

[mol212524-bib-0055] Zhou HL , Luo G , Wise JA and Lou H (2014) Regulation of alternative splicing by local histone modifications: potential roles for RNA‐guided mechanisms. Nucleic Acids Res 42, 701–713. 24081581 10.1093/nar/gkt875 PMC3902899

[mol212524-bib-0056] Zhou Z , Qiu J , Liu W , Zhou Y , Plocinik RM , Li H , Hu Q , Ghosh G , Adams JA , Rosenfeld MG *et al* (2012) The Akt‐SRPK‐SR axis constitutes a major pathway in transducing EGF signaling to regulate alternative splicing in the nucleus. Mol Cell 47, 422–433. 22727668 10.1016/j.molcel.2012.05.014 PMC3418396

[mol212524-bib-0057] Zhu YJ , Zheng B , Wang HY and Chen L (2017) New knowledge of the mechanisms of sorafenib resistance in liver cancer. Acta Pharmacol Sin 38, 614–622. 28344323 10.1038/aps.2017.5 PMC5457690

