# Supplementary figures and images for: Alternative splicing in human cancer cells is modulated by the amiloride derivative 3,5‐diamino‐6‐chloro‐N‐(N‐(2,6‐dichlorobenzoyl)carbamimidoyl)pyrazine‐2‐carboxide
Source: Mol Oncol. 2019 Jun 1;13(8):1744–62. doi: 10.1002/1878-0261.12524 (PMC6670021; doi:10.1002/1878-0261.12524)

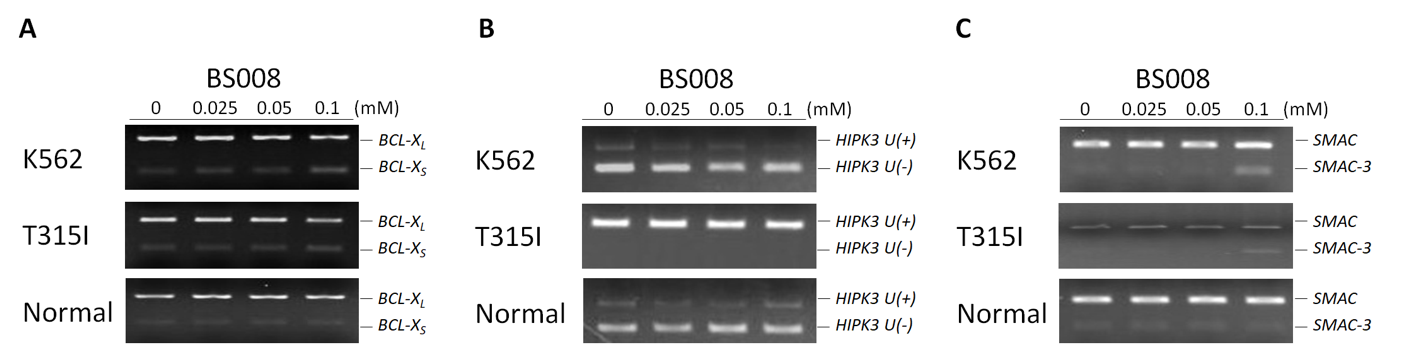

Supplement: Supplementary file 1 — Fig. S1. Effects of BS008 on alternative splicing of (A) BCL‐X, (B) HIPK3, and (C) SMAC RNAs. Messenger RNA was extracted and detected using RT‐PCR for the AS in K562, T315I and normal mononuclear cells. [file MOL2-13-1744-s001.tif]

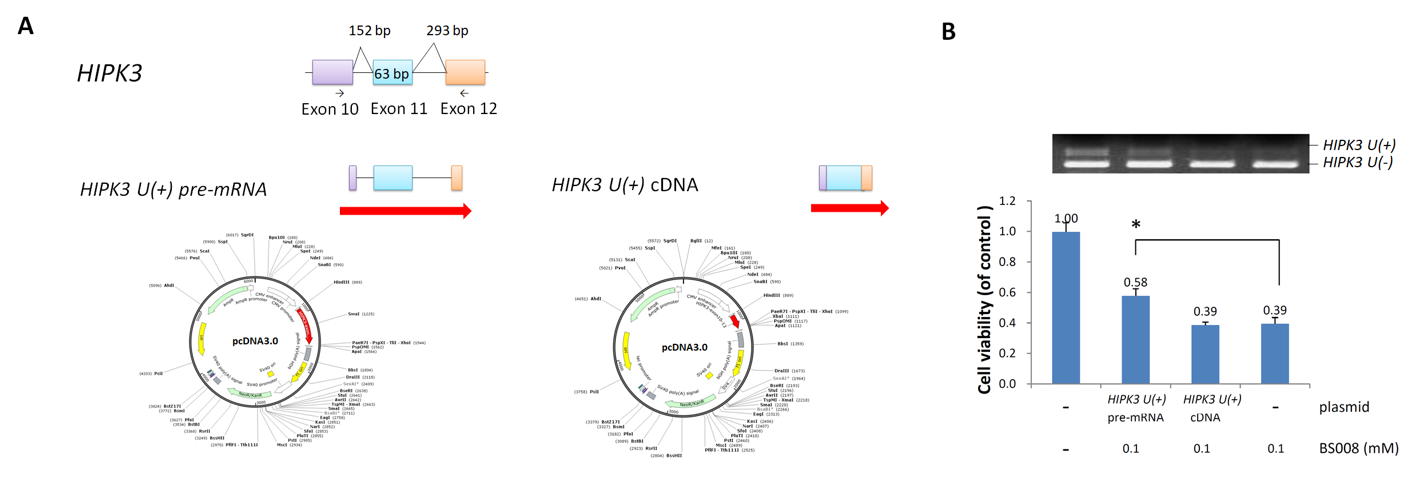

Supplement: Supplementary file 2 — Fig. S2. Effects of HIPK3 splicing on BS008‐induced cell death. (A) Experimental design and vectors. (B) K562 cells were transfected with the expression plasmids as indicated for 24 h prior to BS008 treatment. RT‐PCR validation of endogenous HIPK3 alternative splicing (upper panel). Cell viability was analyzed using MTT assay followed by 24‐h treatment with BS008 (lower panel). Data are presented as mean ± standard deviation from six independent experiments (*P < 0.05 using unpaired two‐tailed Student's t‐test). [file MOL2-13-1744-s002.tif]

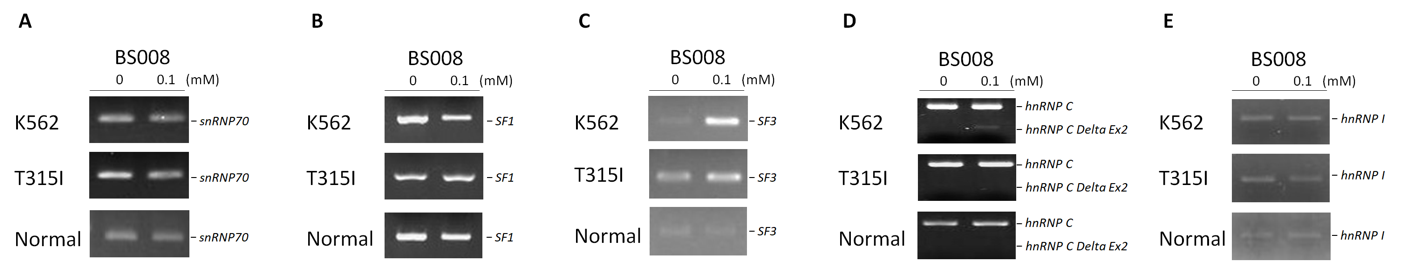

Supplement: Supplementary file 3 — Fig. S3. Effects of BS008 on alternative splicing of (A) snRNP70, (B) SRSF1, (C) SRSF3, (D) hnRNP C, and (E) hnRNP I RNAs. Cells were treated with BS008 for 24 h and then harvested for RT‐PCR analysis. [file MOL2-13-1744-s003.tif]

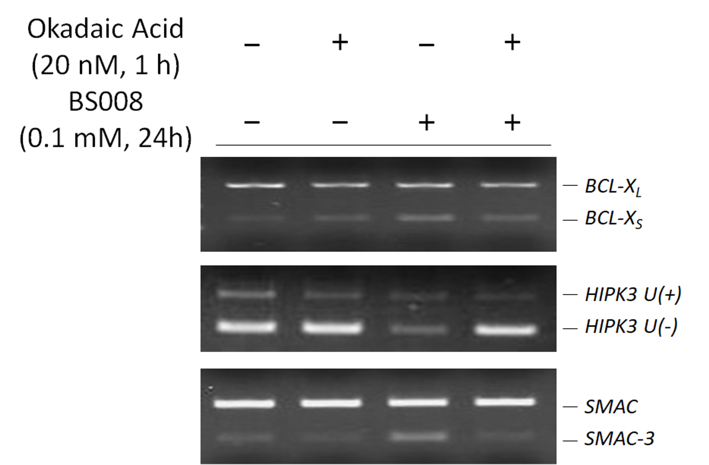

Supplement: Supplementary file 4 — Fig. S4. Effects of PP1 phosphatase on BS008‐induced alternative splicing. RT‐PCR results from K562 cells pretreated with (+) or without (‐) okadaic acid and then exposed to BS008 for 24 h. [file MOL2-13-1744-s004.tif]
