# Supplementary material for: Alternative splicing in human cancer cells is modulated by the amiloride derivative 3,5‐diamino‐6‐chloro‐N‐(N‐(2,6‐dichlorobenzoyl)carbamimidoyl)pyrazine‐2‐carboxide
Source: Mol Oncol. 2019 Jun 1;13(8):1744–62. doi: 10.1002/1878-0261.12524 (PMC6670021; doi:10.1002/1878-0261.12524)
Supplement: Supplementary file 5 — Table S1. Information about the Small interfering RNA used in the study. Table S2. Information about the primers used in reverse transcription polymerase chain reaction. Table S3. Ten known target proteins of amiloride collected from BindingDB. Table S4. Alternative splicing‐related proteins. [file MOL2-13-1744-s005.docx]

**Table S1** Information about the Small interfering RNA used in the study

| Gene | Sequences |
| --- | --- |
| snRNP70 (sense) | GCUCCGGAGAAUGGGUAUUTT |
| snRNP70 (antisense) | AAUACCCAUUCUCCGGAGCTT |
| hnRNP I (sense) | GCACAGUGUUGAAGAUCAUTT |
| hnRNP I (antisense) | AUGAUCUUCAACACUGUGCTT |
| scrambled control (sense) | UUCUCCGAACGUGUCACGUTT |
| scrambled control (antisense) | ACGUGACACGUUCGGAGAATT |

**Table S2** Information about the primers used in reverse transcription polymerase chain reaction

| Gene | Forward primer | Reverse primer |
| --- | --- | --- |
| *AATF* | CAGTGAGGATGATGGTGTGG | CTCCAGCTTCCTCTTTGCAG |
| *AIFM1* | TGCTTTGAAGCAGAAGCTGG | GTTCTGGTGTCAGCCCTAAC |
| *API5* | CAGGCCGACCTAGAACAGAC | AACTCATCTCCGCCAACAAT |
| *ATM* | ATTTGATTGAATTGATGGCAG | CAACTTCCGTAAGGCATCG |
| *BCL-X* | GACGAGTTTGAACTGCGGTA | TGCTGCATTGTTCCCATAGA |
| *HIPK3* | AGCCTGCCACTACCAAGAAA | CAGCAATTTCTTGCCTCTCC |
| *hnRNP C* | AGACGAAGACTGAGCGGTTG | GAGTTCATGGAGCGAGGATC |
| *hnRNP I* | TCTCCGCCATTTTGTGAGTC | TCAGGAGGTTGGTGACCTTC |
| *KDM4A* | TGCACGAGGAAGAAAGGAAG | ACAGTTGGCTCAGAGGCTTG |
| *KDM5A* | TGAGACCTGTGCTTCTGTGG | GTTTCATCCATCATGGCCTC |
| *KDM5C* | AGACCCTCTTGGCTACATCG | GTAGTGGGAGCGTAGCAAGG |
| *KDM7A* | ACCGCTTCATGATCGAGTG | GTCATGTCTGTGCCAGTTCC |
| *NFKB1* | TGGAAGCACGAATGACAGAG | ATTTCCTCCCCTCCAGTCAC |
| *SMAC* | AGTAACCCTGTGTGCGGTTC | CTGCCACACTTCATCTTCCTC |
| *snRNP70* | TCACAATGATCCCAATGCTC | CTCGATGAAGGCATAGCCAC |
| *SRSF1* | GCGGTCTGAAAACAGAGTGG | CTGCTGTTGCTTCTGCTACG |
| *SRSF3* | CAGCCTCACCTCACCATTTC | CACCACTTCTCTTGCAAACTG |

**Table S3** Ten known target proteins of amiloride collected from BindingDB

| Protein name | Uniprot ID |
| --- | --- |
| Serotonin Transporter (SERT) | P31652 |
| Adenosine A1 receptor | P28190 |
| Urokinase-type plasminogen activator (uPA) | P00749 |
| Adenosine receptors; A1 & A2 | P29274 |
| Solute carrier family 22 member 2 | Q9R0W2 |
| Solute carrier family 22 member 1 | Q63089 |
| Vascular adhesion protein 1(VAP-1) | Q16853 |
| Amiloride-sensitive sodium channel alpha-subunit | P37088 |
| Polo-Like Kinase 1 | P53350 |
| Protein Kinase D1 | Q15139 |

*Collected from BindingDB database.

**Table S4** Alternative splicing related proteins

| Protein name | Uniprot ID |
| --- | --- |
| Muscleblind-like protein | Q9NR56 |
| Muscleblind-like protein 2 | Q5VZF2 |
| RNA-binding protein Nova-1 | P51513 |
| Polypyrimidine tract-binding protein 1 | P26599 |
| RNA binding protein fox-1 homolog 1 | Q9NWB1 |
| Splicing factor U2AF 65 kDa subunit | P26368 |
| Steroidogenic factor 1 | Q13285 |
| Serine/arginine-rich splicing factor 1 | Q07955 |
| Serine/arginine-rich splicing factor 2 | Q01130 |
| Serine/arginine-rich splicing factor 3 | P84103 |
| Serine/arginine-rich splicing factor 4 | Q08170 |
| Heterogeneous nuclear ribonucleoprotein A1 | P09651 |
| Heterogeneous nuclear ribonucleoproteins A2/B1 | P22626 |
| RNA-binding protein Raly | Q9UKM9 |
| U1 small nuclear ribonucleoprotein 70 kDa | P08621 |
